# Immunometabolic reprogramming, another cancer hallmark

**DOI:** 10.3389/fimmu.2023.1125874

**Published:** 2023-05-19

**Authors:** Vijay Kumar, John H. Stewart

**Affiliations:** ^1^ Department of Interdisciplinary Oncology, Stanley S. Scott Cancer Center, School of Medicine, Louisiana State University Health Science Center (LSUHSC), New Orleans, LA, United States; ^2^ Louisiana State University- Louisiana Children’s Medical Center, Stanley S. Scott, School of Medicine, Louisiana State University Health Science Center (LSUHSC), New Orleans, LA, United States

**Keywords:** cancer, immunity, inflammation, immunometabolism, immunometabolic reprogramming, TME, TIME

## Abstract

Molecular carcinogenesis is a multistep process that involves acquired abnormalities in key biological processes. The complexity of cancer pathogenesis is best illustrated in the six hallmarks of the cancer: (1) the development of self-sufficient growth signals, (2) the emergence of clones that are resistant to apoptosis, (3) resistance to the antigrowth signals, (4) neo-angiogenesis, (5) the invasion of normal tissue or spread to the distant organs, and (6) limitless replicative potential. It also appears that non-resolving inflammation leads to the dysregulation of immune cell metabolism and subsequent cancer progression. The present article delineates immunometabolic reprogramming as a critical hallmark of cancer by linking chronic inflammation and immunosuppression to cancer growth and metastasis. We propose that targeting tumor immunometabolic reprogramming will lead to the design of novel immunotherapeutic approaches to cancer.

## 1 Introduction

Cancer is the second leading cause of death worldwide as 10 million deaths resulted from cancer in 2020 and 70% of cancer deaths occurred in developing or low-middle-income countries (LMICs). Furthermore, it is projected that the incidence of cancer will increase to 28.4 million cases in 2040 (1). Sub-Saharan countries will witness a 92% cancer increase between 2020 and 2040. Several factors contribute to the rising incidence of cancer in these countries, including environmental pollution, the adoption of western diets, increased alcohol uptake, lack of exercise, and increased tobacco use.

Advances in medicine have now established that cancer cells differ from normal cells in many ways. For example, cancer cells exhibit uncontrolled cell division and proliferation, never mature, ignore signals required for the orderly progression of the cell cycle, cell death (apoptosis), specialization, and shedding. In addition, cancer cells express neoantigens and evade the host’s immune recognition (2, 3). Hence, cancer cells develop intratumoral heterogeneity, including altered cellular architecture/morphology, physiology (including their metabolism), subtypes, and evade cell death and their immune recognition (4–7). Additionally, nuclear compartmentalization (chromatin re-organization) in the tumor microenvironment (TME) regulates the gene expression that controls many processes, including immune cell development and programing, discussed in detail elsewhere (8–10). Furthermore, extrachromosomal DNAs (ecDNAs) are emerging as crucial mediators of cancer pathogenesis, gene regulation and epxression, and emerging treatment resistance (11–14). For example, ecDNAs promote increased oncogene expression and subsequent poor prognosis in many cancers (15–18).

Further development in the field led to the recognition of the six hallmarks of cancers almost 20 years ago (19, 20). Metabolic reprogramming among cancer cells and immune escape were also included later as additional hallmarks (21). Many reviews have further emphasized cancer and immune cell metabolism as a foundation mechanism for tumor immunology (22–27). For example, DePeaux and Delgoffe have discussed in detail the importance of decreasing TME metabolic barriers to increase the efficacy of tumor immunotherapy, including oncolytic viral therapy (OVT) (22). Whereas, Leone and Powell have discussed the metabolism of immune cells, specifically T cells, in the TME and exploiting differential metabolic plasticity for increasing the efficacy of immune checkpoint inhibitors (ICIs) (23). Hence, immunometabolism in the TME is critical in tumor immunopathogenesis, metastasis, and efficacy of existing immunotherapies. Hanahan recently upgraded the list of cancer hallmarks to include canonical and prospective characteristics (28). Different metabolic determinants of tumor initiation have been identified and discussed in detail (29). Therefore, we propose to add tumor-supportive immunometabolic reprogramming to the list of cancer hallmarks. The work herein discusses immunometabolic reprogramming of tumor-infiltrating immune cells as a critical hallmark of cancer progression.

## 2 Immune surveillance failure in cancer

**Figure 1:**
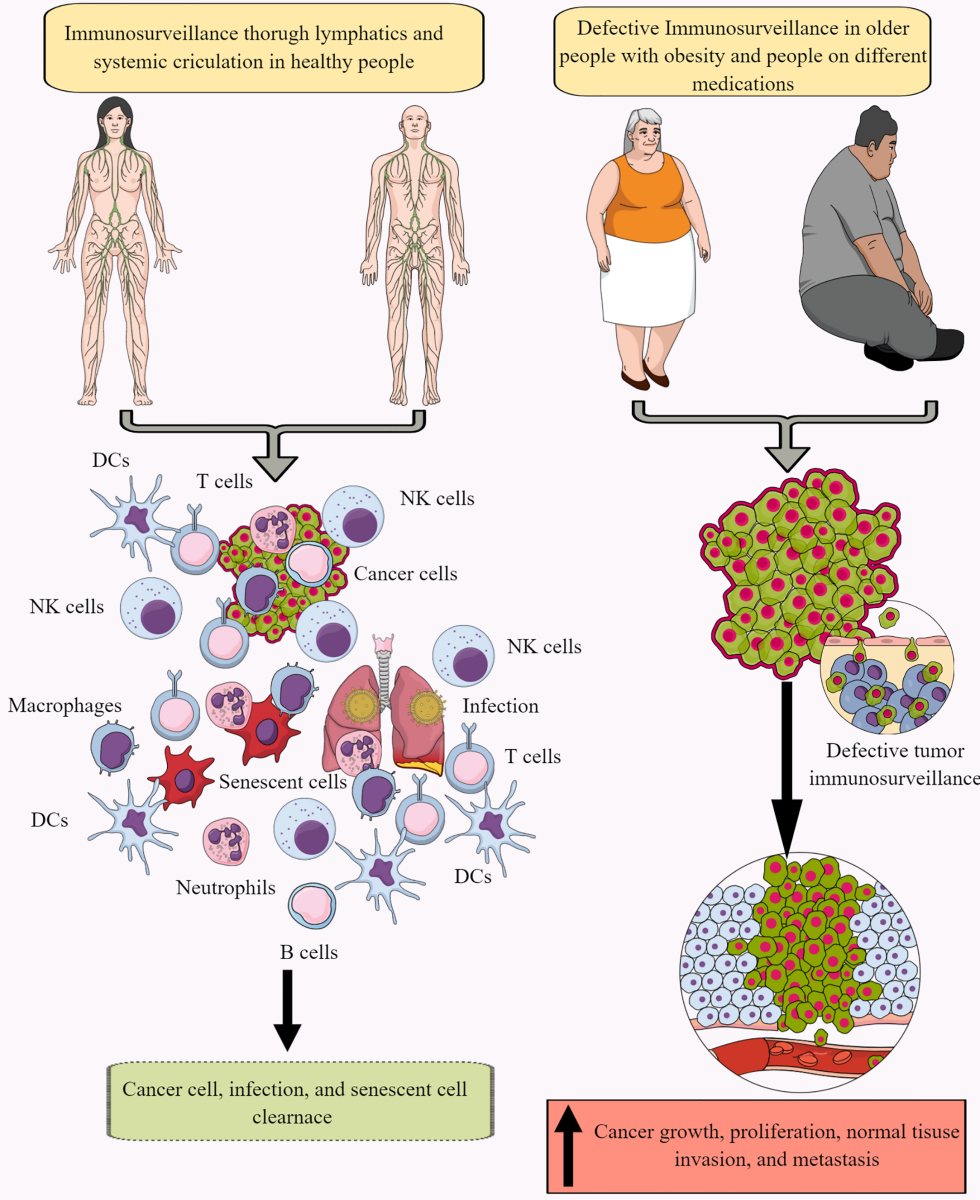
Immunosurveillance and cancer. The continuous immune surveillance of target organs by immune cells (innate and adaptive immunity) through lymphatics and systemic circulation keeps a check on altered or cancer cells in healthy individuals. This helps to maintain homeostasis by removing altered or cancer cells. However, several factors, including aging, obesity, repeated or chronic infections, and different medications, dysregulate or suppress regular immune surveillance leading to a tumor or cancer development. See text for details.

Immune surveillance protects the host from endogenous and exogenous threats, including cancer development, infections, and premature aging (**Figure 1**) (30–34). However, aging and certain medications (antibiotics and antivirals) dysregulate immune surveillance to induce a tumor supportive environment (35–38). The immune system-mediated patrolling and monitoring to prevent cancer is called cancer or tumor immunosurveillance (39, 40). Tumor immune surveillance (immunosurveillance) requires tumor cell-derived molecules, including heat-shock proteins (HSPs) and double-stranded DNA (ds-DNA), which are recognized by pattern recognition receptors (PRRs) (41, 42). For example, CD91 (a receptor for HSP gp96)is crucial in cancer immune surveillance and cancer arising in the absence of CD91 are highly immunogenic (42, 43). However, the tumor microenvironment (TME) supports immunosurveillance escape and therefore supports cancer growth, differentiation, and metastasis (**Figure 1**) (44, 45). For example, TME T cells induce galectin-9 secretion from tumor cells derived from various malignant tumors. The released galectin-9 suppresses the antitumor cytotoxic activity of CD8^+^ T and natural killer (NK) cells (46). Galectin-9 in cancer cells combines with V-domain Ig-containing suppressor of T cell activation (VISTA, an immune checkpoint protein) to support the protumorigenic immunosuppressive TME (46, 47). The transforming growth factor-β (TGF-β) via TGF-β receptors (TGF-βRs) and suppressor of mothers against decapentaplegic-3 (smad-3) protein induce the VISTA expression on cancer and T cells in the TME to promote immunosuppression. TGF-β and VISTA mediate immunosuppression by polarizing naïve T cells to regulatory T cells (T_regs_) and pro-inflammatory M1 macrophages to M2 macrophages by increasing the SNAIL or snail family transcriptional repressor 1 (SNAI1) expression and increasing the myeloid-derived suppressor cells (MDSCs) activity (48–52). Thus, cancer cells and immune cells in the TME coordinate to create a tumor suppressive tumor immune microenvironment (TIME) for the growth, division, and metastasis of cancer cells. Cancer cell metabolism also plays a significant role in escaping from tumor immune surveillance via different mechanisms, including altering immunometabolic reprogramming.

## 3 Metabolic reprogramming among cancer cells in TME

Cancer cells differ from normal cells in maintaining homeostasis regarding their energy demand. Cancer cells undergo metabolic reprogramming to maintain their fastidious growth and proliferation status. For example, they reprogram themselves for rapid adenosine triphosphate (ATP) synthesis to meet increased energy demand, macromolecule synthesis, and tight maintenance of their redox status (53). The cancer cell metabolic reprogramming is crucial for their survival in the stressful TME with its spatially and temporally heterogenous concentrations of glucose, glutamine, and oxygen favoring hypoxia (54). For example, TGF-β in the TME increases aerobic glycolysis via glucose transporters and glycolysis enzymes to meet their high energy demand (55). Additionally, TGF-β also increases TME lactate level, which directly correlates with cancer cell metastasis. Furthermore, the acidic TME supports tumor cell survival, proliferation, and resistance to apoptosis (56–58).

**Figure 2:**
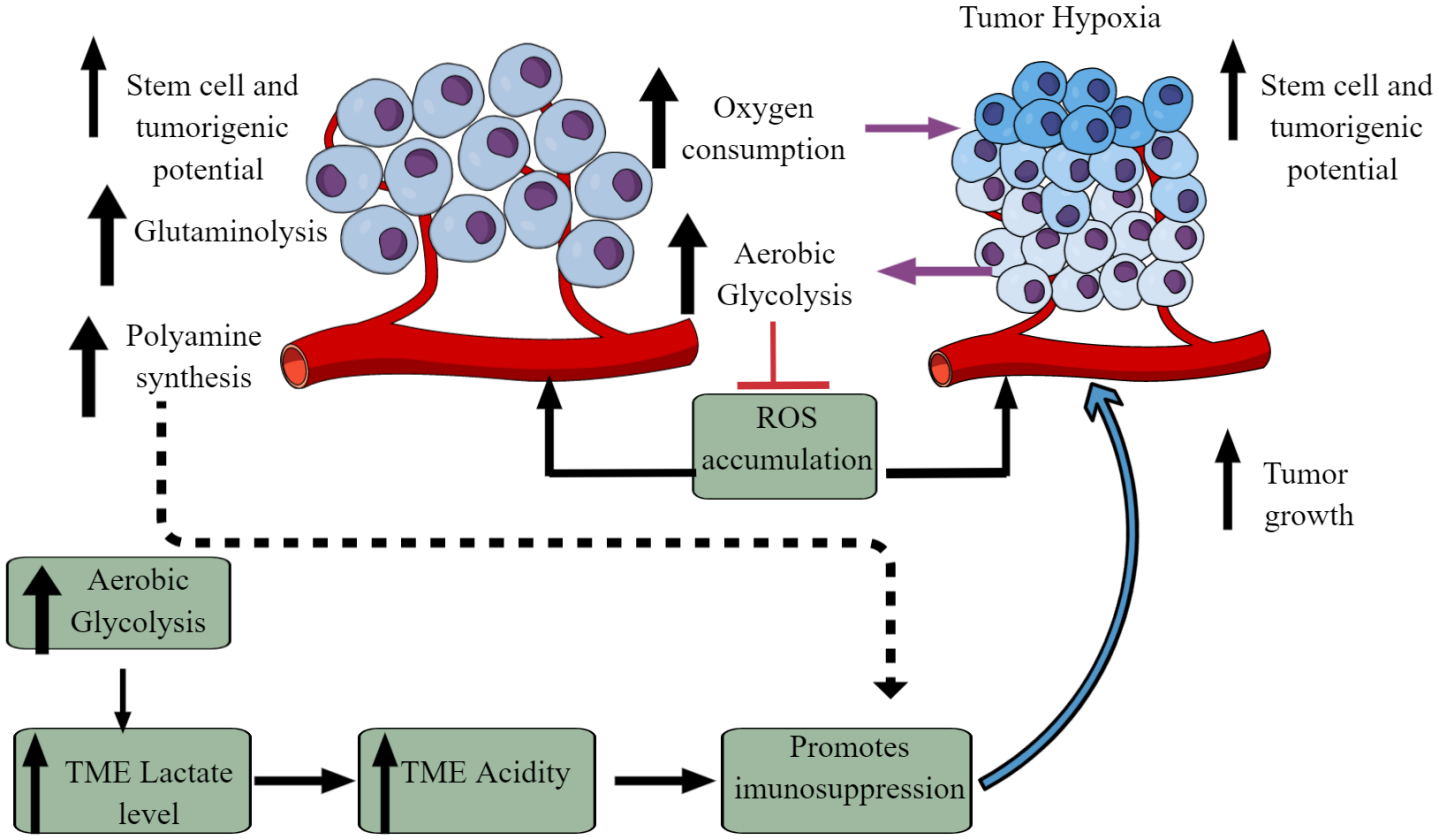
Altered cellular metabolism among cancer cells. Due to the altered physiological and metabolic demands, cancer cells undergo metabolic reprogramming. For example, due to their high energy demand as a response to their fastidious growth and proliferation, cancer cells depend on aerobic glycolysis, causing lactate accumulation and an increased acidic environment. Furthermore, increased aerobic glycolysis elevates oxygen consumption inducing hypoxia. The increased hypoxia and acidity (lactate accumulation) cause immunosuppression to escape from the host immune response. Immunosuppression promotes tumor growth. Additionally, other metabolic mechanisms (polyamine synthesis, glutamine metabolism) also increase in tumor cells, limiting nutrient availability to residential and infiltrated immune cells, causing their immunosuppression. Details are mentioned in the text.

The Warburg effect is an excellent example of cancer cell metabolic reprogramming, shifting from oxidative phosphorylation (OXPHOS) to aerobic glycolysis (**Figure 2**) (59–61). However, the observed Warburg effect in the TME does not depend on oxygen availability and the carcinogenic origin of cancer (54, 62). Hypoxia induces the hypoxia-inducible factor-1α (HIF-1α) that regulates the transcription of at least 60 genes regulating tumor cell survival, growth, proliferation, tumor angiogenesis, invasion/metastasis, glucose metabolism, immune cell function (63–66). High pyruvate dehydrogenase kinase (PDK) activity in tumor cells increases glycolysis. It also suppresses reactive oxygen species (ROS) production and accumulation, enhancing their stem cell and tumorigenic potential (**Figure 2**) (67). The aerobic glycolysis in TME can even occur in the non-dividing cells, indicating that the Warburg effect controls the tumor biomass and enhances their stem cell-like phenotype and oncogenic potential (67). Thus, the increased glucose uptake in tumor cells decreases its concentration in the tumor interstitial fluid (TIF) and increases extracellular lactate levels with increased lactate dehydrogenase (LDH) activity **Figure 2** (68). Tumors expressing nucleus accumbens-associated protein-1 (NAC1) also upregulate LDH-A activity that further supports lactate accumulation in TME (69). The increased lactate level in the TME inhibits antitumor immune responses by T cells, macrophages, and DCs, through different mechanisms, including immunometabolic reprogramming (70–75).

A recent study has provided some of the first experimental evidence of the Warburg effect in patients with cancer (76). For example, clear cell renal carcinoma (ccRC) exhibits increased aerobic glycolysis compared to the adjacent normal kidney, and ccRC has suppressed glucose oxidation compared to tumors of other anatomical sites, including the brain and lungs (76, 77). Hence, ccRC is the first human tumor to demonstrate a convincing shift toward glycolysis, as indicated by the intraoperative ^13^C infusions. It is important to note that the altered metabolic environment in the TME induces a metabolic competition between tumor and immune cells that helps in cancer progression (78, 79). Like glucose metabolism, the increased glutaminolysis in cancer cells also creates a glutamine-deficient tumor immune microenvironment (TIME) for immune cells (**Figure 2**). Tumor cells exhibit the highest glutamine uptake in TME compared to infiltrated immune cells (80). Notably, the increased glutamine uptake suppresses the glucose uptake across tumor-resident cell types, emphasizing that glutamine metabolism suppresses glucose uptake without glucose being a limiting factor in the TME (80). Cancer cells over express the methionine transporter SLC43A2. Therefore, they outcompete CD8^+^ T cells for methionine uptake and utilization (81). The decreased methionine availability to CD8^+^ T cells decreases the methyl donor *S*-adenosylmethionine (SAM), inhibiting dimethylation at lysine 79 of histone H3 (H3K79me2). The loss of H3K79me2 in CD8^+^ T cells decreases signal transducer and activator of transcription 5 (STAT5) expression and alters their cytotoxic action against tumor cells. Furthermore, the methionine utilization by tumor cells in the TME of hepatic cell carcinoma increases T cell exhaustion (82). Thus, strategies to deprive methionine uptake by cancers cells or providing methionine to TME T cells has a potential cell-specific immunometabolic targeting in different solid cancers.

Additionally, increased polyamine biosynthesis and transport occur in tumor cells as indicated by the induction of ornithine decarboxylase (ODC), a hallmark for tumorigenesis (**Figure 2**) (83–86). Polyamines suppress the immune response to promote tumor growth and directly influence it through numerous tumor-supportive mechanisms (**Figure 2**) (87–90). Along with tumor cells, myeloid cells (tumor-associated macrophages (TAMs), dendritic cells (DCs), and MDSCs compete with T cells to utilize polyamines to exert their immunosuppressive action (91). Hence, cancer and immunosuppressive myeloid cells compete with T cells in the TIME for polyamine uptake and utilization. In addition, polyamine metabolism is a central determinant of CD4^+^T cells to differentiate into different functional Th subtypes (Th1, Th2, Th17, and T_regs_). Therefore, polyamine deficiency in CD4^+^T cells results in the failure to adopt a correct subset specification by affecting the tricarboxylic acid (TCA) cycle and histone deacetylation (92). Also, the decreased availability of polyamines to T cells supports their differentiation to immunosuppressive T_regs_ and its targeting reverses the TME immunosuppression (93–96). Thus, cancer cell metabolism alters the TIME via affecting immunometabolic reprogramming.

## 4 Immunometabolism in TIME

Immunometabolism combines classical metabolism and immunology to understand the immune cell phenotype and function by combining immunology and metabolism experimental approaches and paradigms (97). Immunometabolism has two subdisciplines: (1) cellular immunometabolism and (2) tissue immunometabolism. Cellular immunometabolism governs the fate of immune cells. At the same time, tissue immunometabolism includes the governing of tissue and systemic metabolism by immune cells to support the adaptations of the host to the surrounding environment (97, 98). Six major metabolic pathways, including glycolysis, the Krebs’s cycle, fatty acid synthesis (FAS), fatty acid oxidation (FAO), amino-acid (AA) metabolism, and the pentose-phosphate pathway (PPP) regulate immune cell function (99). The details of immunometabolism during inflammation or inflammatory immune cell function have been discussed elsewhere (100–102).

Despite having the maximum capacity to uptake intratumoral glucose, myeloid cells in the TIME shift their immunometabolic reprogramming to tumor-promoting anti-inflammatory, immunosuppressive phenotype such as M2 macrophages, N2 neutrophils, MDSCs, and tolerogenic DCs (80). Hence, nutrient partitioning in the TIME is programmed in a cell-intrinsic manner through mammalian target of rapamycin complex 1 (mTORC1) signaling and the expression of genes related to glucose and glutamine metabolism. For example, glucose deprivation to immune cells prevents their pro-inflammatory tumor suppressive action in the TIME, indicating that tumor cells are still the biggest glucose consumer. Therefore, we will primarily focus on immunometabolic reprogramming among different immune cells that support tumor growth via immunosuppression.

### 4.1 Immunometabolic reprogramming among tumor-resident or infiltrated macrophages to support tumor growth, proliferation, and metastasis

**Figure 3:**
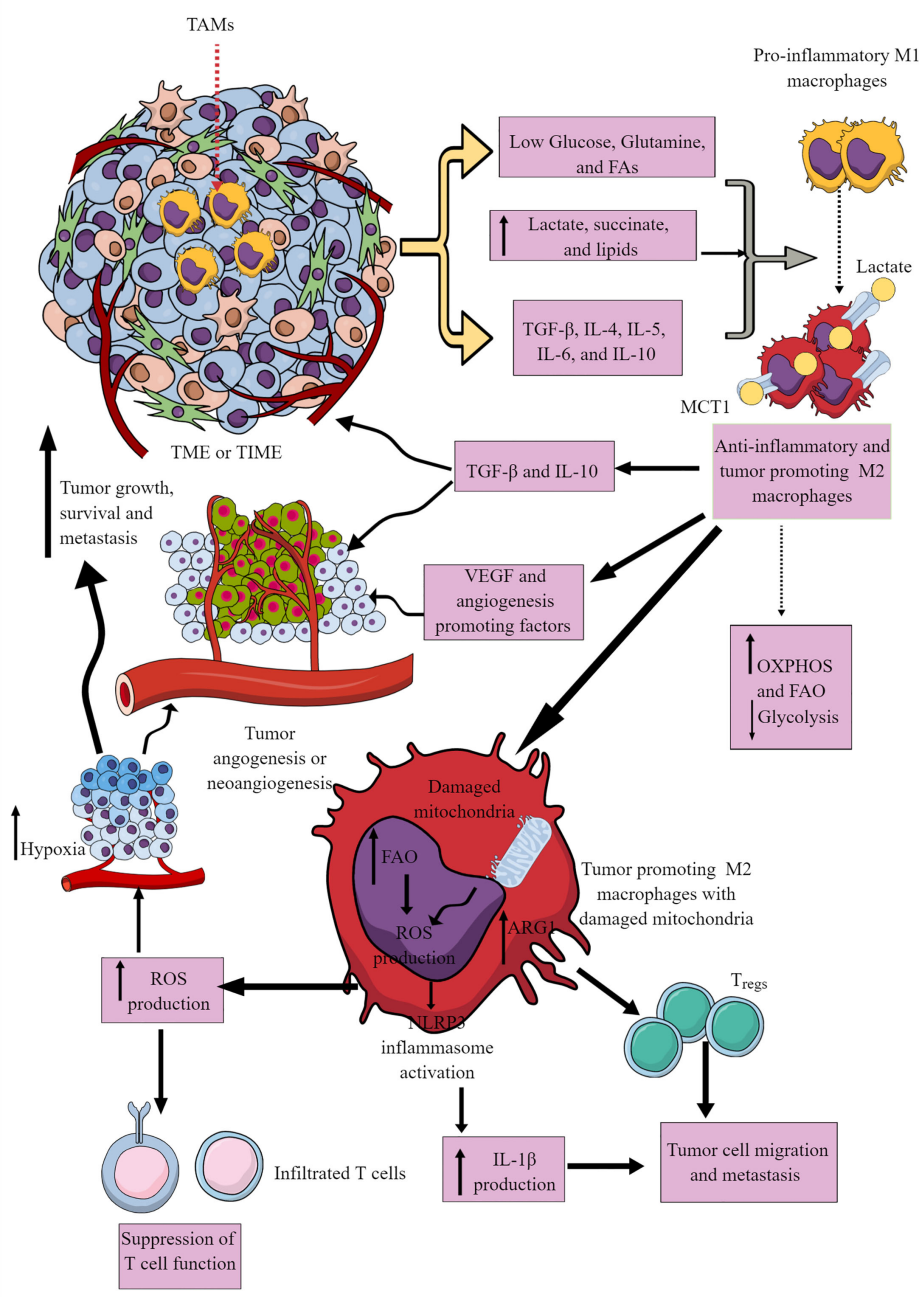
Tumor-associated macrophages (TAMs) in immunosuppressive TME or TIME and their immunometabolic reprogramming. Several factors, including low nutrient availability, increased lactate, succinate, and lipid levels, and different Th2 cytokines (TGF-β, IL-4, IL-5, IL-6, and IL-10) polarize antitumor and pro-inflammatory M1 TAMs to tumor-promoting and immunosuppressive M2 TAMs. These M2 TAMs release immunosuppressive cytokines (IL-10 and TGF-β) to support an immunosuppressive TIME by supporting T_regs_. M1 TAMs undergo immunometabolic reprogramming to polarize to M2 TAMs. For example, M2 TAMs show increased OXPHOS and FAO to survive in the nutrient-deprived TIME or TME. The increased ROS production due to the damaged mitochondria in M2 TAMs suppresses the antitumor T cell immune response. Intracellular ROS in M2 TAMs activates NLRP3 inflammasome to produce IL-1β supporting tumor cell migration and metastasis. The increased ROS production supports TME or TIME hypoxia, supporting tumor angiogenesis, growth, survival, and metastasis. For details, see the text.

Most immune cells are present within the invasive margins and central zone of tumors (103). However, macrophages often comprise the dominant immune cell population in TIME as they include the first pro-inflammatory innate immune cell responders in the chronic inflammatory environment, which later polarize to tumor-supportive immunosuppressive M2 or TAMs (104–106). M1 to M2 macrophages polarization occurs in response to low glucose, glutamine, and FAs availability in a nutrient competitive TME. M2 polarization is further supported by increased TGF-β, IL-4, IL-5, IL-6, and IL-10 availability in the TME (**Figure 3**). TAMs support tumor growth, survival, proliferation, and metastasis by supporting tumor angiogenesis, chemoresistance, and immunosuppression (107–110). Hence, understanding their immunometabolic reprogramming in TME or TIME is warranted.

For example, M1 macrophages depend on aerobic glycolysis to infiltrate the hypoxic TME and exert their pro-inflammatory and anti-tumor actions (98, 111). The IL-4-dependent M1 to M2 macrophage polarization supports OXPHOS through interferon regulatory factor 4 (IRF4) and mTORC2 activation (112). However, IL-4-mediated M2 macrophage polarization does not require immunometabolic reprogramming to FAO (113). Also, the IL-4-mediated M1 macrophage polarization to M2 phenotype only occurs only when NO^·^ generation is blocked due to the dysregulated mitochondrial function (114, 115). TME/TIME and IL-4 synergistically increase protein kinase RNA-like ER kinase (PERK)-signaling cascade in macrophages to promote immunosuppressive M2 transition, activation, and proliferation (116). PERK activation induces phosphoserine aminotransferase 1 (PSAT1) and serine biosynthesis via activation transcription factor-4 (ATF-4). The increased serine biosynthesis supports an enhanced mitochondrial function and α-ketoglutarate (α-KG) synthesis required for Jumonji domain-containing protein-3 (JMJD3)-dependent epigenetic modification (116). On the other hand, PERK activity loss impedes mitochondrial respiration and FAO crucial for M2 macrophages. Hence, the immunometabolic reprogramming among macrophages depends on stimulus, tissue environment, and mitochondria health. TME and associated TIME are complex due to severely altered tumor cell phenotype, function, and different oncometabolites.

The hypoxic and glucose-deprived TME induces regulated in development and DNA damage response 1 (REDD1) on TAMs that suppresses mTORC1 signaling and associated glycolysis (**Figure 3**) (117, 118). The increased levels of other oncometabolites, including lactate and succinate in TME, further support the M1 to M2 macrophages or TAMs polarization (**Figure 3**) through different mechanisms, including yes-1 associated protein (YAP) and NF-κB inhibition via G protein-coupled receptor 81 (GPR-81)-mediated signaling (119, 120). Macrophages in TIME or TME uptake lactate via increased expression of monocarboxylate transporter 1 (MCT1) that increases OXPHOS and FAO to generate M2 macrophages or TAMs (**Figure 3**) (121, 122). There are three types of M2 macrophages (M2a, M2b, and M2c), which secrete common immunosuppressive cytokines (TGF-β and IL-10) and chemokines to support tumor growth (**Figure 3**) (123). Also, TAMs promote angiogenesis via secreting VEGF and other angiogenesis-promoting factors to support tumor growth, proliferation, and metastasis (**Figure 3**) (117–119).

Cancer cells secrete M-CSF that promotes fatty acid synthase (FASN) activity in myeloid cells, including TAMs (124). FASN in TAMs via peroxisome proliferator-activated receptor (PPAR)β/δ activation promotes increased IL-10 synthesis and release. IL-10 promotes immunosuppression, angiogenesis, tumor growth, and metastasis (**Figure 3**). Also, tumor-cell-produced lipids simultaneously orchestrate M1 to M2 macrophage polarization and survival in TME or TIME via inducing ER stress response by reshuffling lipid composition and saturation on the ER membrane (**Figure 3**) (125). Furthermore, ER stress induces inositol-requiring enzyme 1 (IRE1, an endoplasmic reticulum stress sensor)-mediated spliced X-box-binding protein 1 (XBP1) production and STAT3 activation. The IRE1 production and STAT3 activation support M2 macrophage polarization and immunosuppressive TIME development (125–127). Hence, conditions favoring M2 macrophage transition exert a strong push towards OXPHOS in TAMs, which damages their mitochondria, producing increased ROS (**Figure 3**) (128). The increased ROS production further supports hypoxia and angiogenesis in TME, adding to tumor growth and metastasis. ROS further suppresses the antitumor action of infiltrated T cells (**Figure 3**). FAO-dependent ROS generation activates NLRP3 inflammasome to release IL-1β from TIME M2 macrophages, supporting tumor cell migration and metastasis (**Figure 3**) (129). Also, exosomes released from tumor cells in TME support the M1 to M2 macrophage transition via activating NLRP6/NF-κB pathway to support immunosuppressive TIME and cancer cell metastasis (130). Arginase 1 (Arg1) expression in TAMs lowers the L-arginine availability to T cells in TME or TIME. It recruits immunosuppressive T_regs_ to support tumor growth and development (**Figure 3**) (131). The simultaneous Arg1 and inducible nitric oxide synthesis (iNOS) expression in TAMs (M1/M2 phenotype) at low arginine concentration may favor ROS and RNS production that may inhibit antitumor T cell function in TIME (132–134).

Also, TAMs show a decreased receptor-interacting protein kinase 3 (RIPK3, a central factor in necroptosis) that inhibits caspase 1 (CASP1)-mediated cleavage of PPAR-γ to support FAO (135). The M2 macrophage polarization also involves increased glutamine catabolism (glutaminolysis) and UDP-GlcNAc-associated modules (136). The increased glutaminolysis replenishes the TCA cycle in immunosuppressive TAMs (137). Thus, the glutamine deprivation or N-glycosylation inhibition decreases M2 polarization and CCL22 production and promotes their polarization to M1-like macrophages (136, 138). The indoleamine 2,3-dioxygenase (IDO) expression in M2 macrophages also increases, which depletes local tryptophan via generating immunosuppressive kynurenine metabolites (139, 140). Hence, immunometabolic reprogramming among TAMs (highest in number among TIME immune cells) gives them an immunosuppressive phenotype. These immunosuppressive macrophages suppress other immune cells, including T cells through direct interaction or secreting immunosuppressive metabolites, switching their immunometabolism to immunosuppressive or exhausted phenotype (141–145).

### 4.2 Neutrophils and Myeloid-derived suppressor cells immunometabolism in TIME

**Figure 4:**
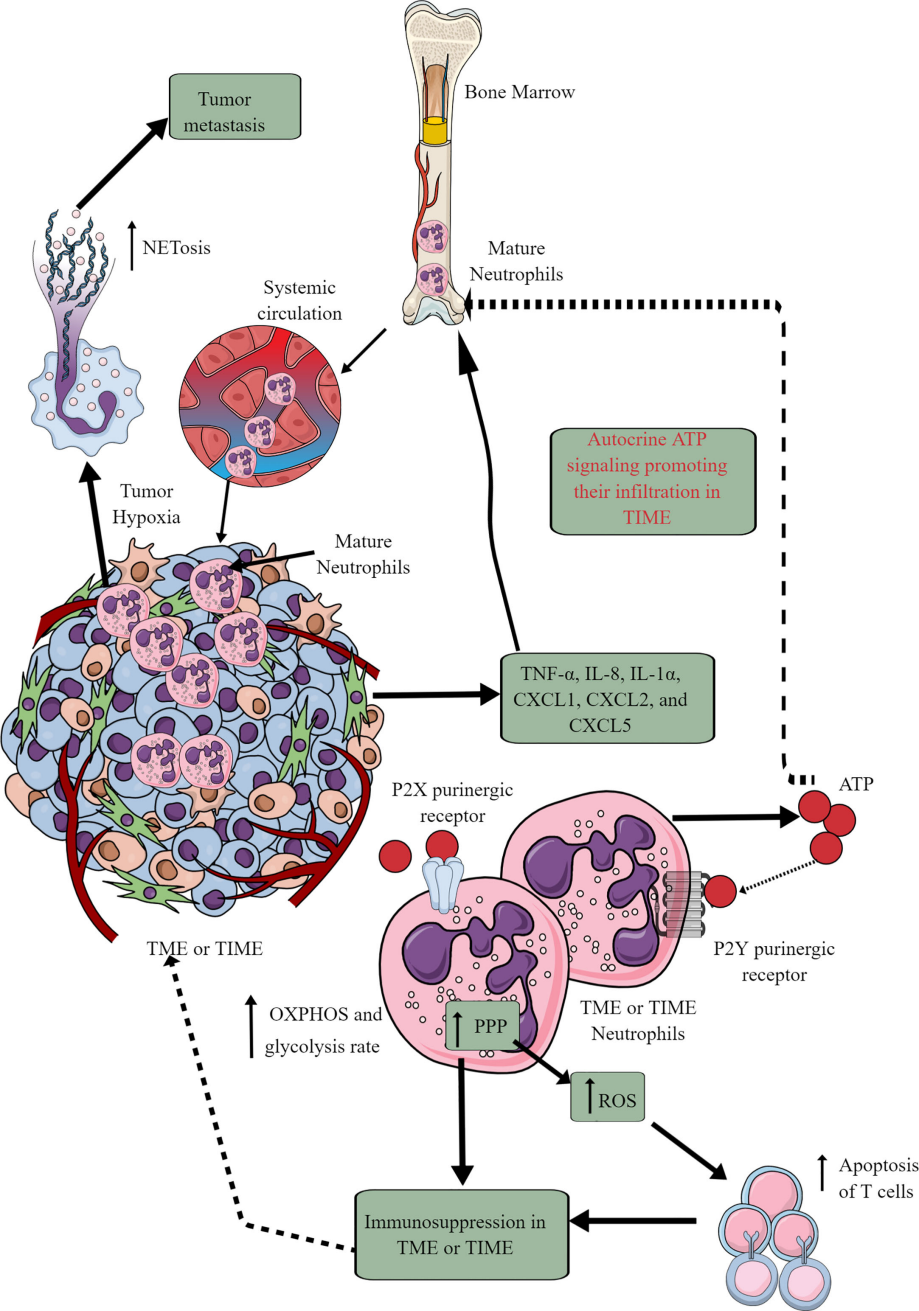
Neutrophils in TME or TIME and their immunometabolic reprogramming. The systemic neutrophil number increases in tumor patients. This increase is due to the increased neutrophil generation in the bone marrow (BM), causing increased infiltration in the TME or TIME. Although only mature neutrophils leave the BM, TIME contains both immature and mature neutrophils. Therefore, different chemokines and cytokines released from TME or TIME cells send the signals to BM for neutrophil chemotaxis. Additionally, ATP released from tumor-associated neutrophils (TANs) acts in an autocrine manner via P2Y purinergic receptors to further support their chemotaxis in TME or TIME. TANs show an increased rate of OXPHOS and glycolysis along with an elevated PPP. The ROS released from TANs induced apoptotic cell death among infiltrated antitumor T cells, causing immunosuppression. Hypoxia in TME or TIME causes NETosis that further supports tumor metastasis. See the text for details.

Tumor cells and immune cells release several factors, including TNF-α, IL-8, IL-1α, CXCL1, CXCL2, and CXCL5 to stimulate neutrophil chemotaxis to the TME (146, 147). Although only mature neutrophils leave bone marrow (BM) for the circulation and target organs, TIME also harbors immature neutrophils (**Figure 4**) (146, 148). At initial stages, neutrophils exert antitumor action but become tumor and metastasis supportive later. They can be classified as antitumor N1 neutrophils that are supported by IFN-β and hepatocyte growth factor (HGF) and protumor N2 neutrophils that are supported by TGF-β and G-CSF (147). The complex immunological functions of neutrophils and their targeting in cancer are discussed elsewhere (147, 149–151).

In cancer-bearing mice, neutrophils leaving the BM show more spontaneous migration than in typical tumor-free mice (152). For example, these neutrophils lack immunosuppressive action, having increased OXPHOS and glycolysis rate than neutrophils of typical tumor-free individuals (**Figure 4**). The aggravated autocrine ATP signaling supports the increased neutrophil infiltration to TIME via purinergic receptors (**Figure 4**) (152). The hypoxic environment in the TME or TIME increases HIF-1α and HIF-2α levels (65). HIF-1α increases neutrophil survival via supporting glycolysis (OXPHOS is not crucial for neutrophils) at initial stages, creating a chronic pro-inflammatory environment to support tumor progression (153). At the same time, HIF-2α increases the lifespan of pro-inflammatory neutrophils called tumor-associated neutrophils (TANs) (154). Also, the PPP in neutrophils supports increased ROS generation that induces apoptotic cell death among infiltrated T cells to support further a tumor suppressive TIME (**Figure 4**) (155, 156). PPP is also involved in the neutrophil extracellular trap (NETs) formation or NETosis by fueling NADPH oxidase with NADPH to produce superoxide that supports cancer metastasis (157). However, immunosuppressive mediators, including TGF-β released at later stages of the tumor, polarize antitumor N1 TANs to pro-tumor N2 TANs (158–160). Also, the glutamine and proline uptake in immature low-density neutrophils (iLDNs) supports their pro-metastasis action inducing NETosis under hypoxic and glucose-deprived conditions (**Figure 4**) (161, 162). NETs promote cancer growth, progression, and metastasis and provide a protective shield to them through different mechanisms discussed somewhere else (163).

**Figure 5:**
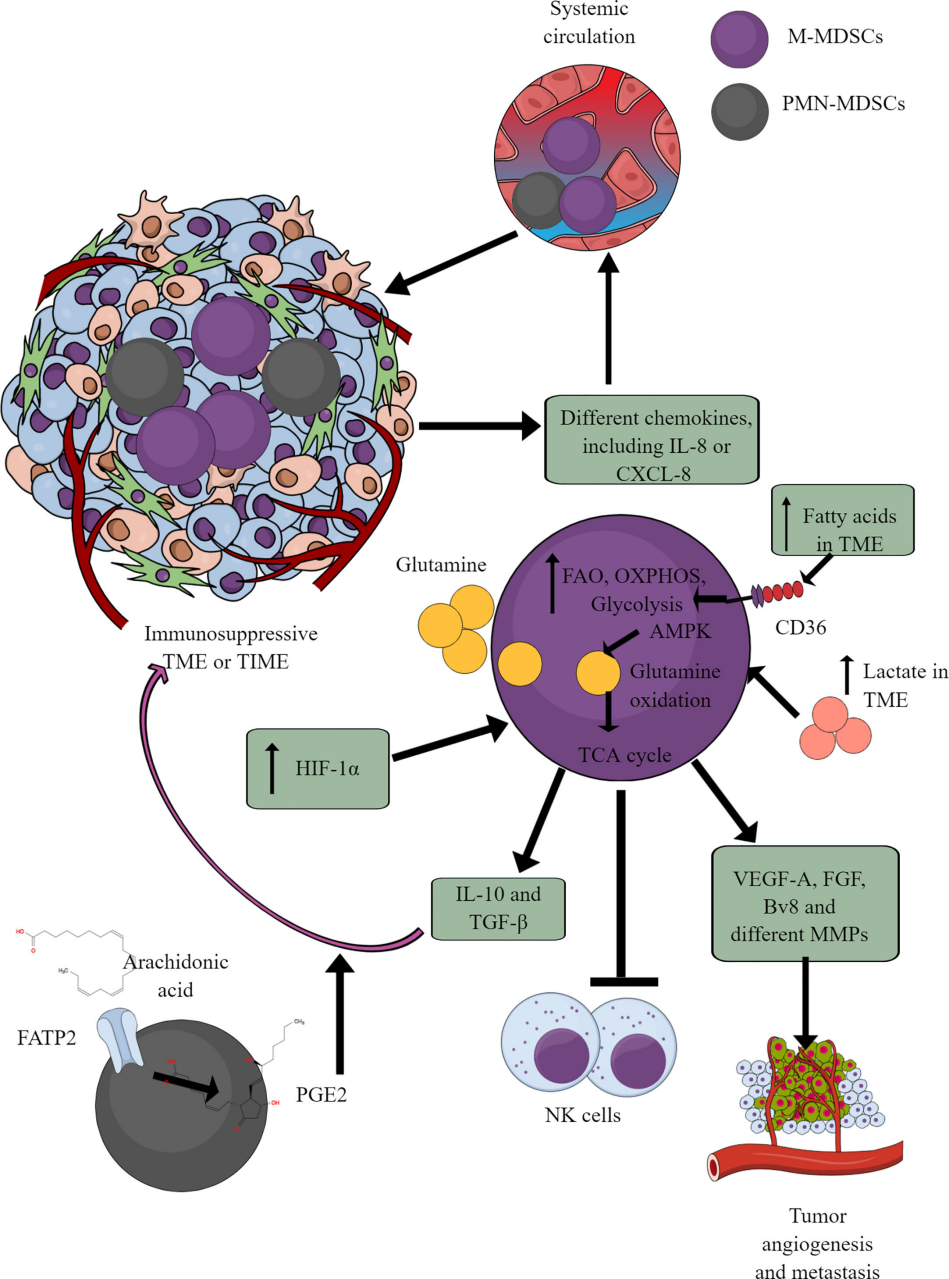
MDSCs in TME or TIME and their immunometabolism. MDSCs infiltration in TME or TIME supports the immunosuppressive microenvironment. IL-8 and many other TME or TIME-released chemokines support their infiltration. To exert their immunosuppressive function, MDSCs show an increased FAO, OXPHOS, and glycolysis. AMPK increase induces glutamine oxidation to support the TCA cycle. The increased lactate level in TME or TIME favors the immunosuppressive function of MDSCs. For example, MDSCs release immunosuppressive cytokines (TGF-β and IL-10), suppress cytotoxic NK cell activity and promote tumor angiogenesis, growth, proliferation, and metastasis. Additionally, arachidonic acid (AA) metabolism to PGE2 in PMN-MDSCs further supports immunosuppressive TIME.

MDSCs are well-known immunosuppressive innate immune cells found only in pathological conditions, including cancer (164–166). They are of two types (1) monocytic-MDSCs or M-MDSCs, and (2) polymorphonuclear-MDSCs or PMN-MDSCs (167). Hence, MDSCs are the pathological phenotypes of neutrophils and monocytes accumulating in pathological lesions, including TME or TIME (164, 165). PMN-MDSCs of patients with cancer also show an increased spontaneous migration characteristic and are present at very early cancer stages (152, 168). Different chemokines, including IL-8 (CXCL8) and CXCR4 chemoattract (in response to miR-494) MDSCs to TME or TIME (**Figure 5**) (169–171). They secrete different immunosuppressive cytokines, including IL-10 and TGF-β, responsible for their immunosuppressive function to support tumor growth, proliferation, neoangiogenesis, and metastasis (170, 172). MDSCs also secrete vascular endothelial growth factor (VEGF)-A, fibroblast growth factor (FGF), and Bv8 (prokineticin or PK), and different MMPs to promote tumor growth and metastases (**Figure 5**) (173, 174).

MDSCs depend on AMPK and FAO for their immunosuppressive function (175, 176). Glutamate or L-glutamine (L-Gln) taken by MDSCs in TME or TIME is oxidized in an AMPK-dependent manner to support their immunosuppressive function by regulating the TCA cycle (**Figure 5**) (177). Even tumor-infiltrated/associated MDSCs (T-MDSCs) synthesize their L-Gln and with increased transglutaminase (TGM) expression that supports their immunosuppressive function and tumor metastases (178, 179). T-MDSCs show an increased FAO, OXPHOS, and glycolysis due to an increased lipid/FAs content in TME or TIME (**Figure 5**) (176). However, the increased FAs in TME or TIME promote FAO in MDSCs via CD36-mediated FA uptake, and FAO inhibition suppresses their immunosuppressive function in TME (176, 180, 181).

The fatty acid transport protein 2 (FATP2) on PMN-MDSCs through arachidonic acid (AA) uptake and prostaglandin E2 (PGE2) synthesis also support the immunosuppressive function of MDSCs (**Figure 5**) (182, 183). Furthermore, the PGE2-mediated negative feedback loop FATP2 and receptor-interacting protein kinase 3 (RIPK3, A negative regulator of FATP2) promotes PMN-MDSCs’ immunosuppressive function (184, 185). GM-CSF controls the FATP2 overexpression on PMN-MDSCs in TIME via STAT5 activation. TME or TIME hypoxia increases the immunosuppressive function of T-MDSCs by increasing the HIF-1α level (**Figure 5**) (186, 187). Furthermore, HIF-1α, along with promoting their immunometabolic reprogramming to immunosuppressive phenotype, also increases the PD-L1 expression that suppresses the cytotoxic and immune-promoting functions of CD8^+^ and CD4^+^T cells in TIME (188). A high lactate level in TME increases the survival and proliferation of immunosuppressive MDSCs through G protein-coupled receptor 81 (GPR81)/mTOR/HIF-1α/STAT3 pathway (189–191). Also, the increased TME lactate level increases the number and proliferation of MDSCs, which inhibit NK cell cytotoxicity (NKCC) (**Figure 5**) (190). Hence, hypoxic TME or TIME supports MDSCs’ immunometabolic reprogramming to FAO to favor their tumor and metastasis-supportive function.

### 4.3 DCs and their immunometabolic reprogramming in TME/TIME

**Figure 6:**
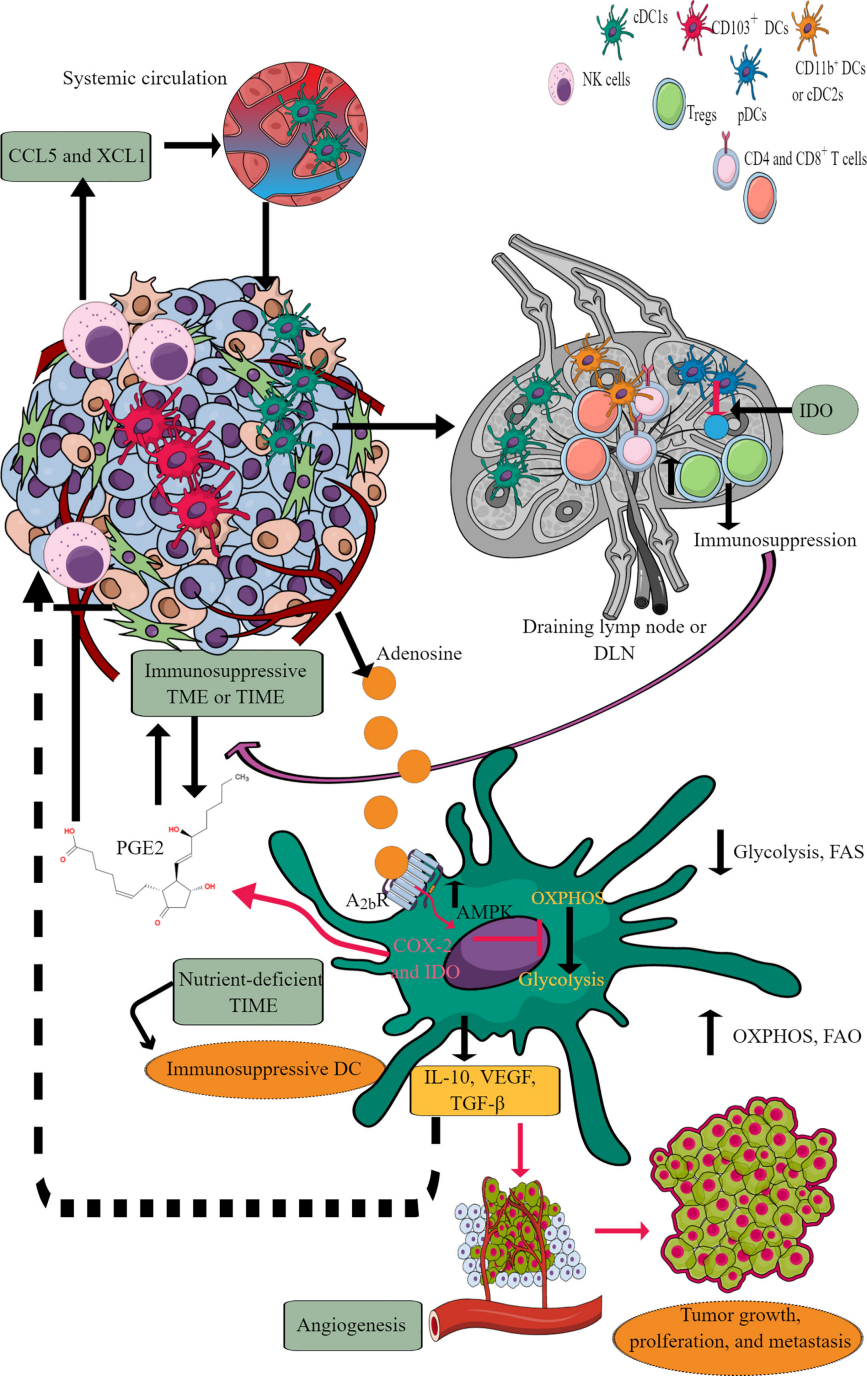
DCs in TIME and their immunometabolic reprogramming. TME or TIME-released chemokines induce DC chemotaxis. cDCs migrate to tumor DLNs for antigen presentation for adaptive immune cells (T and B cells) to induce antitumor immunity. However, IDO release from pDCs induces immunosuppression. Furthermore, adenosine in TME or TIME via A2bR blocks immunometabolic shift to glycolysis from OXPHOS and increases AMPK levels. Thus, tumor-associated DCs (TADCs) show an increased OXPHOS and FAO giving them an immunosuppressive phenotype to survive in the nutrient-deficient TME or TIME. These immunosuppressive TADCs release different factors and molecules to support immunosuppressive TIME, angiogenesis, and tumor growth and metastasis. Details are mentioned in the text.

DCs are potent antigen-presenting cells (APCs), which play a crucial role in generating and regulating immune response via recognizing different pathogens and inflammogens and presenting antigens to adaptive immune cells (T and B cells) (192). They also serve a part of first responding innate immune cells against cancer via antigen presentation despite constituting a rare immune cell population (CD103^+^DCs) within TME or TIME capable of activating CD8^+^T cells (**Figure 6**) (193, 194). Conventional DCs (cDCs) at early malignancy recognize dying tumor cells and migrate to draining lymph nodes (DLNs) to present tumor antigens to CD4^+^ and CD8^+^ T cells (195, 196). For example, type 1 cDCs (cDC1s) prime cytotoxic CD8^+^T cells, and type 2 cDCs (cDC2s) activate antitumor helper CD4^+^T cells (197–199). The antitumor action of cDC1s in TIME depends on NK cells as they release cDC1 chemo-attractants CCL5 and XCL1 to bring them in (**Figure 6**) (200, 201). However, the prostaglandin E2 (PGE2) release by tumor cells in TME or TIME suppresses NKCC and the production of cDC1 chemo-attractive chemokines (**Figure 6**). Thus, cDC1s lose their antitumor function due to the evasion of the NK cell-cDC1 axis and other immune cells with tumor growth. Furthermore, cDC2s (CD11b^+^DCs) in tumor DLNs also express PDL-1 and suppress T cell-mediated antitumor immunity (**Figure 6**) (202, 203). Additionally, monocyte-derived DCs (mo-DCs) with pro-inflammatory properties comprise another type of DCs populating tumors (198). Also, the plasmacytoid DCs (pDCs) in tumor DLNs release IDO that directly activates mature T_regs_ to create an immunosuppressive TIME (**Figure 6**) (204). The details of immunologic and immunoregulatory functions of DCs in TME or TIME are mentioned elsewhere (205–208). We will focus their immunometabolic reprogramming in TME or TIME.

Under a steady state, DCs depend on OXPHOS for their energy demand to maintain immune homeostasis (209). For example, bone marrow-derived DCs (BMDCs) depend on FAO for OXPHOS to meet the energy demand, but the involvement of FAO for OXPHOS in cDCs and pDCs is not yet clear (192, 209). FAO and OXPHOS do not provide the maximum threshold for DCs to secrete cytokines and activate T cells to create a pro-inflammatory environment. The pro-inflammatory PRRs, like toll-like receptor-4 (TLR-4) stimulation, reprograms DC immunometabolic state from OXPHOS to glycolysis within minutes, like other myeloid immune cells (209–211). The shift from OXPHOS to glycolysis induces their antigen presentation potential through increased major histocompatibility complex (MHC)-I and -II expression, co-stimulatory molecules (CD80 and CD86), and cytokine synthesis and release. Although increased glucose uptake by DCs during the early stages of activation is accompanied by lactate production, this does not reflect a commitment to Warburg metabolism as a mechanism for ATP production because, during this time, ATP is provided by OXPHOS (211). Instead, glycolysis fulfills the citrate needs of DCs is filled by glycolysis (211). The export of citrate from the mitochondria into the cytoplasm through the citrate transporter SLC25A is significant for fueling FAS required for activated DCs to increase the size of critical organelles (Golgi bodies and endoplasmic reticulum or ER) involved in protein synthesis and secretion. Intriguingly, the enlargement of these compartments co-occurs with increased gene expression downstream of TLRs but is regulated post-transcriptionally by increased glycolytic flux. This is controlled by the Akt-dependent phosphorylation and subsequent activation of hexokinase II (essential to catalyze the first step of glycolysis) (211).

The Akt activation involves TANK-binding kinase 1 (TBK1)/I-kappa-B kinase epsilon (IKKϵ), activation downstream of RIG-I–like receptor (RLR), indicating that the rapid glycolysis is a typical response to any innate immune recognition by DCs. This Akt activation occurs regardless of PI3K or mTOR (two canonical Akt upstream activators) inhibition (210, 211). Different PRRs, including TLR2, TLR6, TLR9, Dectin-1, and -2 activation, induce immunometabolic reprogramming to glycolysis in DCs that governs their inflammatory status and motility (212, 213). Notably, early glycolysis induction in DCs occurs independently of their pro-inflammatory phenotype. This allows DCs to rapidly respond metabolically to these danger signals originating in the TME (211).

DCs fail to mature in the absence of OXPHOS to glycolysis transition. Also, DCs showing weak inflammatory response lack long-term glycolytic reprogramming requiring increased glycolytic gene expression (212). Thus, a prolonged and increased glycolysis enzymatic gene expression is crucial for maintaining pro-inflammatory DCs and their migration. Also, DCs utilize pre-existing glycogen stores to support shifting from OXPHOS to glycolysis during their inflammatory stimuli to drive their TLR-dependent activation (214). The glycogenolysis inhibition attenuates TLR-mediated DC maturation and impairs their ability to act as APCs. Therefore, it is likely that even weak inflammatory signals can induce early glycolytic reprogramming through glycogenolysis without a significant and prolonged gene transcription crucial for glycolysis reprogramming. However, this is not true for other myeloid cells, including macrophages, which depend on external glucose supply through glucose transporter 1 (Glut1) upon inflammatory stimuli. Thus, only strong pro-inflammatory signals can induce prolonged inflammatory phenotype and DC motility in LNs.

IL-10 and AMP-activated protein kinase (AMPK, the central regulator of catabolic pathways and OXPHOS) inhibit glycolysis (215). The FAS inhibition enhances DCs’ capacity to activate allogeneic and Ag-restricted CD4^+^ and CD8^+^ T cells and induce CTL responses (216). Further, FAS blockade increases DC expression of Notch ligands and enhances their ability to activate NK cell immune phenotype and IFN-γ production. ER stress enhances DC’s immunogenic function upon FAS inhibition, accounting for its higher immunogenicity (216). Conversely, the ER stress lowering by 4-phenylbutyrate (4-PBA) suppresses their increased immunogenic action due to FAS inhibition. TLR7/8 stimulation with promoter-associated RNA (pRNA) increases FAO and OXPHOS in human mo-DCs due to branched-chain alpha-keto acid dehydrogenase complex E1-alpha subunit (BCKDE1α) phosphorylation in a phosphatase and tensin homolog (PTEN)-induced putative kinase 1(PINK1)-dependent manner. Interestingly, inducing PINK1 activity in tolerogenic DCs stimulates FAO and renders them immunostimulatory (217).

Tumor-associated DCs (TADCs), like tumor-associated T cells, also face the harsh nutrient-deficient environment that activates AMPK, inhibiting the immunometabolic reprogramming from OXPHOS to glycolysis. For example, AMPK supports OXPHOS by upregulating proliferator-activated receptor γ co-activator (PGC-1α) that binds to PPAR-γ to promote mitochondrial biogenesis, oxidative metabolism and antagonize anabolic metabolism (218, 219). Thus, TADCs lose their APC properties and migration capacity to DLNs to prime and induce a robust adaptive immune response against tumor antigens. The recognition of exogenous adenosine monophosphate (AMP) by adenosine A_2b_ receptor expressed on DCs, including TADCs, upregulates their pro-tumorigenic functions, including angiogenesis via releasing VEGF, TGF-β, and creating an immunosuppressive environment through releasing IL-10 and expressing cyclooxygenase-2 (COX-2) and IDO (220–223). IDO (IDO1 and IDO2) activity metabolizes tryptophan (an essential amino acid) into kynurenine (224). Thus, the tryptophan depletion activates a stress response kinase called general control non-derepressing 2 (GCN2) in T cells that inhibits their proliferation and biases naïve CD4^+^T cells to develop into FoxP3^+^T_regs_ (225–227). Also, the kynurenine and other metabolites bind to the aryl hydrocarbon receptor (AhR) on T cells, promoting their differentiation to T_regs_ along with supporting the immunosuppressive macrophage and DC phenotype (226, 228–230).

Catabolism of pre-existing glycogen in DCs is crucial to initiate glycolysis independent of external glucose supply in response to the TLR activation (214). However, in TME or TIME, the continuous TLR signaling, including the TLR9 activation in response to the host cell-derived DNA creates an immunosuppressive TIME due to the increased IDO expression (231–233). Furthermore, TLR9 ligand CpG ODN 2006 is a poor adjuvant to induce CD8^+^T cells responsible for clearing tumor cells (234). This may be due to the poor DCs activation or their suppression through IDO generation. Further studies are required in this direction. The increased AMPK expression in TADCs also transforms them into tolerogenic DCs due to increased FAO and OXPHOS (210, 235). Furthermore, the aberrant lipid accumulation in TADCs due to the transport of extracellular lipids via macrophages scavenger receptor 1 (MSR1) diminishes their antigen-presenting capacity that suppresses their adaptive immune activation property to fight against tumors (236, 237). Also, the tumor-released Wnt5 molecule triggers PPAR-γ activation through β-catenin, which activates FAO by upregulating carnitine palmitoyltransferase-1A (CPT1A, a fatty acid transporter) in TADCs and induces a tolerogenic phenotype and secrete IDO to create an immunosuppressive TIME by upregulating T_regs_ (238, 239). Furthermore, the Wnt5 also blocks the immunometabolic shift to glycolysis in TADCs and induces an increased FAO. In addition, β-catenin induces vitamin-A metabolism in TADCs and FAO to produce retinoic acid (RA), further promoting T_regs_ generation to create an immunosuppressive TIME (240) Thus, TME or TIME DCs also become potent immunosuppressive immune cells and lose their antigen presentation characteristics to further support adaptive immunity against tumors due to their immunometabolic reprogramming supporting their survival but not potent immune function.

### 4.4 Immunometabolic reprogramming among innate lymphoid cells, including NK cells in TIME

ILCs are a relatively new class of immune cells, which phenotypically appear as adaptive lymphoid cells. However, they are lineage negative and do not express antigen-specific receptors encoded by rearranged genes, including T cell or B cell receptors (TCRs or BCRs). ILCs also do not show V(*D*)J recombination required for somatic hypermutation (SHM)/recombination, like T and B cells (241). However, they respond to various immunogenic stimuli, including pathogens, to mounting a pro-inflammatory immune response. Additionally, they are highly localized to mucosal surfaces (gastrointestinal, reproductive, and respiratory tracts). The details about different types of ILCs, including ILC1s or group 1 ILCs (NK cells and helper ILC1s), ILC2s (group 2 ILCs, produce Th2 cytokines), and ILC3s (group 3 ILCs, include RORγt^+^ ILCs and lymphoid tissue inducer or LTi cells) inflammation and their interaction with adaptive immune cells have been discussed elsewhere (242–246).

**Figure 7:**
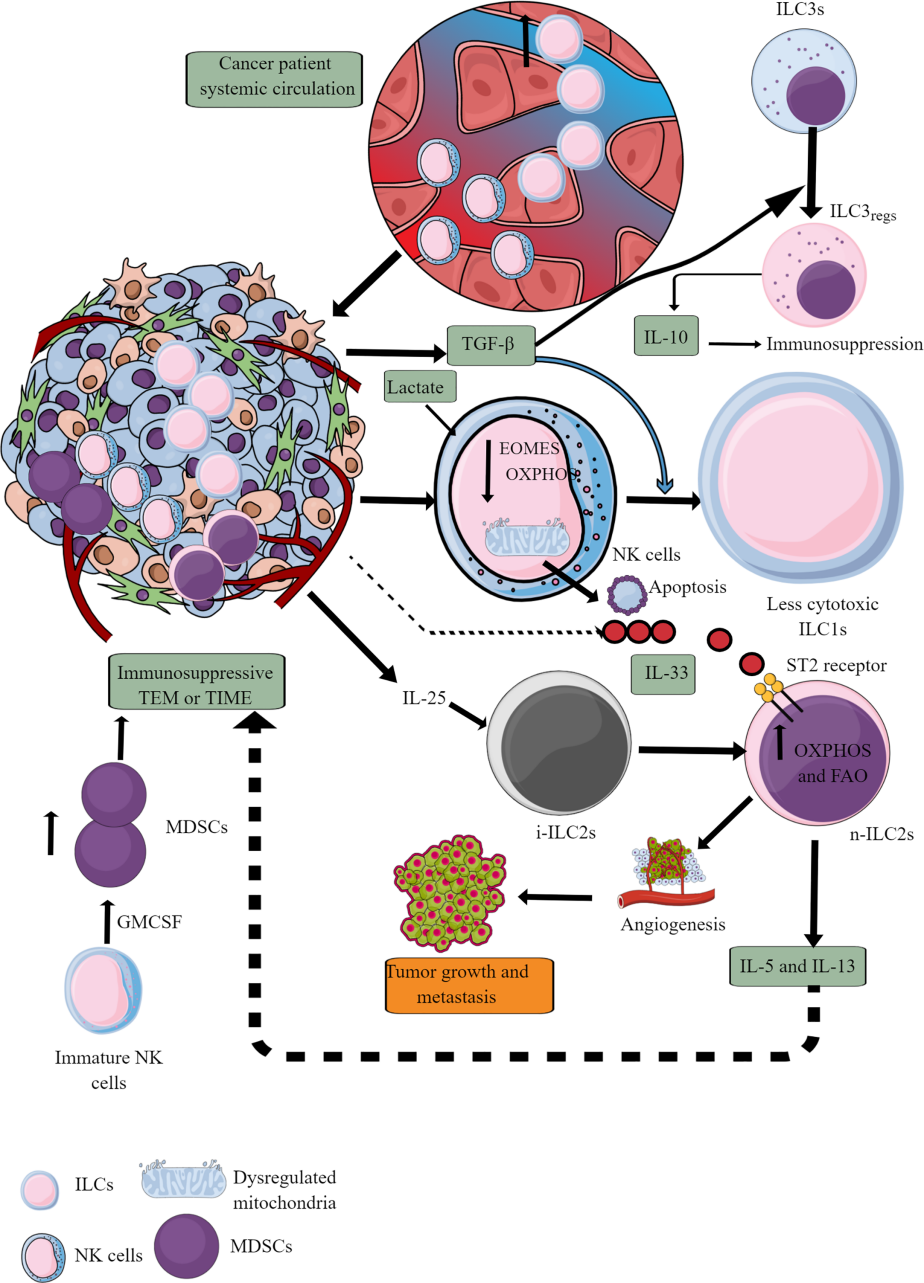
ILCs and NK cells in TIME and their immunometabolism. Different ILCs, including cytotoxic NK cells, are present in TME or TIME. The increased TGF-β levels in TIME transform high cytotoxic NK cells to less cytotoxic ILC1s and ILC3s to ILC3regs, which release IL-10 to support immunosuppressive TIME. Also, the high lactate levels in TIME or TME decrease NK cell OXPHOS, induce mitochondrial damage, and their apoptosis. Hence, the NK cell cytotoxicity (NKCC) is blocked in the immunosuppressive TIME that supports tumor growth. Furthermore, i-ILC2s polarize to n-ILC2s in the presence of TIME IL-25, further supporting angiogenesis, tumor growth, and metastasis by releasing immunosuppressive cytokines (IL-5 and IL-13). IL-5 and IL-13 are released from n-ILC2s in response to IL-33, increasing their OXPHOS and FAO. GM-CSF release from immature NK cells increases MDSCs proliferation, supporting immunosuppression. See text for details.

ILCs increase in the circulation of patients with cancer compared to healthy controls, indicating that they also infiltrate TME or TIME of different cancers (**Figure 7**) (247–251). Patients with a high number of circulating ILCs, including NK cells with great cytotoxic action, are less prone to develop cancer and metastasis (252–254). The ILC (NK cells, ILC1s, ILC2s, and ILC3s) infiltration into the TME at the early (premalignant) stage induces anti-tumor TIME to kill tumor cells through different mechanisms, including direct cytotoxic action and recruitment of different immune cells, including cytotoxic T cells, and eosinophils (255–259). The details of ILCs, including NK cells in early TME, have been discussed elsewhere (260, 261).

At later stages, NK cells infiltrating TME become less cytotoxic ILC1s (inefficient in controlling the growth and metastasis of tumor cells) in the presence of TGF-β secreted by tumor cells and other immunosuppressive immune cells (262–264). TGF-β also downregulates eomesodermin (EOMES) or T-box brain protein 2 (Tbr2) expression (**Figure 7**) (262). EOMES and T-box protein in T cells (T-bet) are crucial for NK cell development, maturation, and cytotoxic function (265–267). Also, EOMES is crucial for invariant NK (iNK)T cell development and differentiation in the thymus and their differentiation to memory-like KLRG1^+^iNKT cells in the periphery (268). iNKT cells facilitate the potent anticancer cytotoxic action of CD8^+^T cells by presenting different lipid and glycolipid antigens to expressed MHC class I-like molecule CD1d, in addition to direct killing (269, 270). iNKT cells also release IFN-γ that further supports tumor cell killing by NK cells (270). Hence, it will be novel to study the impact of TGF-β on iNKT cell development and function in TME or TIME, which depends on EOMES expression. Furthermore, TGF-β in TME also reprograms otherwise antitumor ILC3s to tumor-promoting regulatory ILC3s (ILC_regs_) and secrete IL-10 (271).

IL-25, an IL-17 cytokine subfamily member in TME or TIME, transforms inflammatory ILC2s (iILC2s) to natural ILC2s (nILC2s) or ILC3-like cells to create an innate tumor-permissive microenvironment through activating ILC2s via inducing IL-17 expression (272, 273). iILC2s have a low RORγt expression, but nILC2s do not (274). Also, these tumor infiltrating ILC2s are highly IL-25R^+^ (273). These nILC2s secrete large amounts of IL-5 and IL-13 (Th2 cytokines), creating an anti-inflammatory or immunosuppressive TIME (272). However, IL-25 exerts a tumor regulatory role through different mechanisms, including eosinophil and B cell infiltration, apoptosis, and Th2 cytokines secretion in TME to create an immunosuppressive TIME (275). The therapeutic blockade of IL-25R in colorectal cancer (CRC) lowers the tumor burden and activates an anti-tumor immune response in mice (273). These ILC2s join IL-25R^+^ MDSCs to create an immunosuppressive TIME in different cancers (276–278). Another study has shown that blocking IL-25 (released from gastrointestinal tuft cells) suppresses gastric cancer in mice, and the ILC2 axis, which is responsible for immunosuppressive IL-13 release (279). IL-33 (a member of IL-1 cytokine family) also promotes tumor survival and progression through different mechanisms, including T_regs_ functional stabilization (280, 281). Also, IL-33 exerts tumor supportive action via regulating PPAR-γ-mediated IL-4, IL-13, and IL-15 (Th2 cytokines) release from ILC2s (**Figure 7**) (282). Thus, antitumor functions of ILCs, including NK cells, ILC2s, and ILC3s, reprogram to tumor-promoting immune activity governed by their immunometabolic reprogramming.

ILCs, including NK cells recruited to the nutrient-competitive TME with tumor cells, adjust their immunometabolic requirement affecting their antitumor immune function. For example, NK cells depend on glycolysis and OXPHOS for their energy requirement under immune homeostasis due to their limited energy or biosynthetic demand (283, 284). Under inflammatory conditions due to increased energy demand to perform a cytotoxic function and cytokine release, immunometabolic reprogramming shifts more towards aerobic glycolysis than OXPHOS, although an increase in OXPHOS also occurs like effector CD8^+^T cells that depends on mTORC1 activation (285–287). However, TME does not support their increased glucose demand to exert their antitumor action. For example, increased TGF-β in TME induces NK cell suppression through decreasing mitochondrial metabolism, including OXPHOS, which is crucial to maintain its high metabolic demand to maintain its antitumor activity (288). This process occurs independently of mTORC1 inhibition. However, TGF-β blocks IL-15-dependent NK cell proliferation and maturation via inhibiting mTOR signaling (289). Thus, it will be interesting to delineate factors responsible for a differential effect of TGF-β on mTOR signaling and dependent metabolic reprogramming, as mTORC1 signaling is crucial for NK cell maturation and proliferation in patients with metastatic cancers (290).

It is important to note that blocking TGF-β restores the anti-tumor function (including metastasis prevention) of NK cells via restoring their immunometabolic reprogramming crucial for cytotoxicity and IFN-γ release (288, 289). Additionally, lactate accumulation in TME also blocks NK cells’ OXPHOS via inducing mitochondrial dysfunction due to increased ROS release, making them energy deficient and causing their apoptosis (**Figure 7**) (291). Thus, it will be interesting to delineate that to escape from apoptosis of TIME NK cells in the presence of TGF-β to polarize to less cytotoxic ILC1s having less energy demand to survive. Also, GM-CSF in TME converts immature NK cells to MDSCs, helping in cancer progression and metastasis (292).

The immunometabolic reprogramming of ILC2s is complex compared to other immune cells. For example, they use OXPHOS and branched-chain amino acids (valine, leucine, and isoleucine) to fuel their polarized mitochondria at their steady state during homeostasis (293). However, their developmental maturation depends on the HIF-1α-glycolysis axis (294). Hence, OXPHOS, branched amino acids, and glycolysis are crucial to maintaining ILC2s’ immune homeostatic function by regulating development and maturation. The release of IL-4, IL-6, and IL-13 (Th2 cytokines) from ILC2s is maintained by increased glutaminolysis, glycolysis, mTOR activation, and FAO (295, 296). However, they continue to OXPHOS through amino acid uptake to maintain their cellular fitness and proliferation (296). The increased FAO takes place in ILC2s of nutrient (glucose and glutamine)-deficient TME or TIME, which reprograms their antitumor function to tumor-promoting via releasing Th2 cytokines causing immunosuppression and angiogenesis (**Figure 7**) (297).

Furthermore, the increased IL-33 level in TME or TIME promotes ILC2’s pro-tumor function via binding to its cognate receptor ST2, promoting temporary storage of externally acquired FA in lipid droplets to make cell membranes (298). These accumulating lipid droplets transform into phospholipids to promote ILC2s proliferation. An enzyme called diacylglycerol o-acyltransferase 1 (DGAT1) regulates this process. PPAR-γ, a key transcription factor, governs this immunometabolic reprogramming crucial for lipid uptake, metabolism, and ILC2 function (297, 298). For example, genetic deletion or pharmacological inhibition of PPAR-γ and DGAT1 in ILC2s blocks the IL-33-mediated cancer growth and metastasis (282). The IL-33-mediated optimal immunometabolic reprogramming in ILC2s also requires ROS, and its inhibition can prevent its tumor-promoting role by suppressing IL-5 and IL-13 release (299). Thus, TME supports immunometabolic reprogramming among ILC2s to create an immunosuppressive TIME that supports tumor growth and metastasis.

### 4.5 Immunometabolic reprogramming among T cells in the TIME

**Figure 8:**
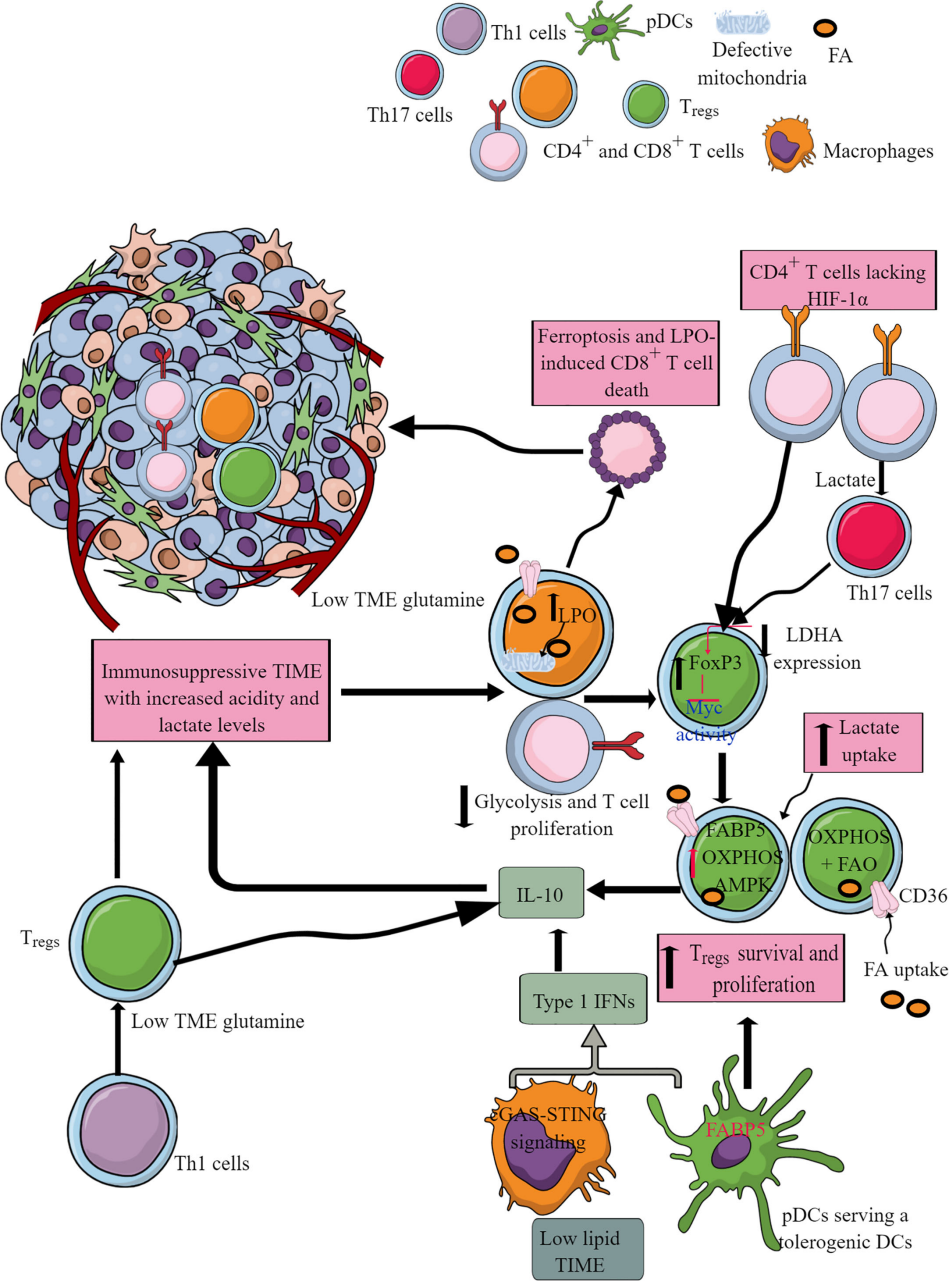
T cell subtypes and their immunometabolic reprogramming in the immunosuppressive TME or TIME. Different Th1 and cytotoxic T cells infiltrate initially to care for growing or premalignant tumors. However, the nutrient-deprived TME or TIME and the presence of immunosuppressive myeloid cells alter their function, including immunometabolic reprogramming. For example, CD8^+^ cytotoxic T cells undergo cell death, including ferroptosis. Additionally, Th1 and Th17 cells in the high lactate and nutrient-deprived (glucose and glutamine) TIME polarize to immunosuppressive T_regs_. All these events support T cell immunometabolic reprogramming to their tumor-supportive phenotype and function, including CD8+T cell death. Details are mentioned in the text.

T cells are crucial adaptive immune cells, which have the potential to regulate the immune system through helper T (Th) cell phenotype and direct killing of tumor cells through their cytotoxic action (CD8^+^T cells) (102). The pro-inflammatory T cells (Th1, Th2, and Th17 phenotypes collectively called T effector (T_eff_) phenotype) depend more on increased glycolysis than OXPHOSS (300, 301). The aerobic glycolysis controls T_eff_ function, including the IFN-γ release through binding the glycolysis enzyme glyceraldehyde 3-phosphate dehydrogenase (GAPDH) to AU-rich elements within the 3’ untranslated region (3’ UTR) of IFN-γ mRNA (302). Also, the lactate dehydrogenase A (LDHA) induction in T cells supports aerobic glycolysis but supports IFN-γ release or Th1 differentiation independent of 3’UTR through epigenetic mechanisms (303). In addition, in acidic TME (due to lactate accumulation), LDH converts lactate to pyruvate and lowers nicotinamide adenine dinucleotide (NAD^+^) levels. The decreased NAD^+^:NADH further blocks glycolysis in T cells (**Figure 8**) (304). The increased lactate level in TME inhibits NAD^+^-dependent GAPDH and 3-phosphoglycerate dehydrogenase (PGDH) activity crucial for NADH reduction and serine production, important for T cell proliferation (**Figure 8**) (304). Serine supplementation rescues T cell proliferation in high lactate TME.

LDHA maintains a high acetyl-coenzyme A (acetyl-CoA) level that promotes histone acetylation and IFN-γ transcription. LDHA deletion in T cells suppresses their IFN-γ-mediated pro-inflammatory action and induces their differentiation to FoxP3^+^ T_regs_. FoxP3 expression in T_regs_ reprograms their immunometabolism to OXPHOS via suppressing Myc activity and glycolysis and increasing NAD^+^ oxidation (**Figure 8**) (305). Due to this, T_regs_ resist highly acidic (lactate) TME and grow and proliferate (306). T_regs_ also take more lactate than T_effs_ to utilize it as a fuel for the TCA cycle or gluconeogenesis, which decreases their glucose need in the highly nutrient-competitive TME (305, 307). T_regs_ highly express lactate transporter, monocarboxylate transporter 1 (MCT-1), for lactate uptake in TME (307). Furthermore, TME lactate induces programmed cell death protein 1 (PD-1 or CD279) expression in T_regs,_ and PD-1 inhibition strengthens T_regs_ in TIME, causing treatment failure with PD-1-based checkpoint inhibitors (308, 309). However, a co-treatment with anti-PD-1 and a LDH inhibitor serves as a better anticancer treatment as this approach inhibits lactylation of Lys72 in MOESIN (a member of the ERM (ezrin, radixin, and moesin) proteins). The MOESIN lactylation inhibition improves MOESIN interaction with TGF-β receptor I and downstream SMAD (suppressor of mothers against decapentaplegic) family member 3 (SMAD3) signaling activating FoxP3 in T_regs_ (310).

T_reg_ differentiation in TME or TIME depends on the Basic leucine zipper transcription factor, ATF-like or BATF transcription factor (311). T_regs_ are highly dependent on FAO or β lipid oxidation and OXPHOS for their immunoregulatory function due to the lower Glut1 and higher AMPK expression (300, 301). The fatty acid binding protein 5 (FABP5, a cellular chaperone long-chain FAs) in T_regs_ regulates OXPHOS and immunosuppressive function by inducing the IL-10 release in response to the type 1 IFN (released in response to cGAS-STING signaling) in low lipid availability TME for immune cells (**Figure 8**) (312). Hence, FABP5 is a gatekeeper for mitochondrial integrity modulating T_regs_. Furthermore, FABP5 expression in pDCs in TME or TIME supports their tolerogenic role via supporting the generation of T_regs_ (**Figure 8**) (313).

T_regs_ generation do not need mTOR kinase (314). Also, the CD36 expression increases in T_regs_, supporting their survival and proliferation in TME or TIME via increased FA uptake (**Figure 8**) (315). Furthermore, CD36 fine-tunes mitochondrial fitness via PPAR-β signaling to increase T_regs_ survival in a lactate-rich acidic TME by increasing OXPHOS (**Figure 8**) (315, 316). On the other hand, CD36 expressed on CD8^+^ cytotoxic T cells increases oxidized lipids/low-density lipoproteins (oxLDLs) uptake that increases lipid peroxidation (LPO) (**Figure 8**) (317). LPO activates p38 mitogen-activated protein kinase (p38MAPK) that induces CD8^+^T cell dysfunction through defective mitochondrial biogenesis in mTOR-independent signaling pathway governing their autophagy and glycolysis (**Figure 8**) (317, 318). CD36-mediated lipid uptake by CD8^+^T cells in TME also causes their ferroptosis and LPO to cause their death and immunosuppression (**Figure 8**) (319). Thus, CD36-mediated FA uptake determines T cell-dependent immunosuppressive TIME. Also, death/damage-associated molecular proteins (DAMPs) in TME promoting chronic inflammation can activate T_regs_ TLRs that, with FoxP3, balance mTORC1 signaling and glucose metabolism to control their proliferation and immunosuppressive function (320, 321). Hence, TME and TIME support T_regs_ for tumor growth and metastasis and induce resistance to chemotherapies and checkpoint inhibitors through immunometabolic reprogramming.

Th17 cells selectively express HIF-1α governed by mTOR signaling, a central regulator of cellular metabolism (301). HIF-1α is crucial for glycolysis induction and maintenance. The lack of HIF-1α in T cells at their differentiation stage reprograms them to develop into T_regs_ (**Figure 8**) (301). The tumor-associated Th17 cells with low glycolysis capacity reprogram to FoxP3^+^T_regs_ (**Figure 8**) (322). Thus, the local tissue environment, including metabolic status, is crucial determines T cell differentiation to their different phenotypes and function.

Low extracellular lactate promotes immune cell infiltration and proliferation at the premalignant stage, including T cells at the site to create a pro-inflammatory TIME to clear tumor cells. For example, CD8^+^T cells under physiologic normoxia utilize glycolysis to exert antitumor action, including IFN-γ release and cytotoxicity (323). The prolyl-hydroxylase (PHD) proteins are intrinsic oxygen-sensing molecules that promote T_regs_ growth and proliferation during hypoxia that develops at later stages of cancer (323). The T cell-specific internal deletion or pharmacological inhibition of PHD increases the antitumor action of tumor-infiltrating T cells. The increased energy demand among tumor cells reprograms their metabolism to increased glycolysis and creates a hypoxic TME. The increased glycolysis among tumor cells in TME increases extracellular lactate accumulation, which impairs the nuclear factor of activated T-cells (NFAT) activation and IFN-γ production by T and NK cells (324–326). This impairs the anticancer/tumor action of tumor infiltrating CD4^+^ and CD8^+^ T cells in the pro-inflammatory environment. For example, tumor infiltrating T cells in the glucose-deprived TME could not reprogram their immunometabolism to glycolysis, forcing them to rely on OXPHOS without exhibiting the T_eff_ phenotype that causes their mitochondrial depolarization and exhaustion (327–329). IL-12 treatment rescues T cell exhaustion by increasing their mitochondrial potential and reducing their dependence of glycolysis (330). Hence, IL-12 treatment prevents forced OXPHOS while maintaining the balanced glycolysis and OXPHOS to maintain their full effector function.

The increased PD-1-PD-L1 signaling (TAMs, cancer cells, and tolerogenic DCs express PD-1 and PD-L1), altered epigenetic reprogramming, and nutrient-deprived stressful TME through coordinating with the TCR signaling prove lethal to tumor-infiltrating CD8^+^T cells by altering their immunometabolic reprogramming (131, 327, 331, 332). Thus, low access to appropriate nutrients (glucose, glutamine, and lipids) imposes a significant barrier to T_effs_ via metabolic stress (333–336). For example, T cells under hypoxic conditions with limited glucose conditions exhibit mTORC1 signaling pathway inhibition, decreased antigen-induced expression of genes (including cell adhesion molecules, cell cycle progression), and CD8^+^T cell proliferation and effector function (335, 337).

The insufficient glucose level in TME or TIME induces apoptosis among T_effs_ *_via_* activating pro-apoptosis genes/proteins, including phorbol-12-myristate-12 acetate-induced protein 1(MAIP1/Noxa, a Bcl2 family protein) and Bcl-2-associated X protein (Bax), destabilizing myeloid cell leukemia 1 (Mcl1), an antiapoptotic Bcl-2 family protein (338). However, memory T_effs_ are not programmed to upregulate FAS, OXPHOS, and reductive glutaminolysis in limited glucose conditions, including TIME, which allows them to maintain their function in the nutrient-limited/depleted microenvironment (339). Thus, naïve T cells survive the nutrient-depleted TME or TIME but lose their effector function, but only memory T_effs_ survive and function in the environment. In addition, increasing FAO activity in CD8^+^T cells in TME or TIME can enhance their cytotoxic action as they show an increased PPAR-α signaling and FA catabolism, which preserves their cytotoxic action (340, 341). However, it should be noted that tumor progression also increases co-inhibitor expression on CD8^+^T cells, and PD-1 blockers delay tumor progression by affecting tumor-infiltrating lymphocyte (TIL) metabolism and function.

The cell motility is controlled by subtype-specific transporters called MCT1 (Slc5a12 and Slc16a1), specifically expressed on CD4^+^ and CD8^+^ T cells. The lactate accumulation suppresses the cytotoxic action of CD8^+^T cells and promotes the CD4^+^T cells switching to Th17 cells (**Figure 8**) (324). Also, IL-2 (a cytokine critical for antitumor T cell function) signaling-mediated STAT5 activation becomes limited in a highly acidic TME (342). This further suppresses antitumor CD8^+^T cell function. The tumor-associated Th17 cells reprogram to FoxP3^+^T_regs_ in TME. The genetic targeting of LDHA in tumors decreases the pyruvate to lactate conversion restoring T and NK cell infiltration and their antitumor cytotoxic function (325). Pyruvate dehydrogenase kinase 1 (PDHK1) via inhibiting PDH determines the cytosolic lactate levels in T cells that varies with T cell subtype (343). For example, Th17 cells show a robust PDHK1 expression, whereas T_regs_ have it at an intermediate level and Th1 cells have very little PDHK1. Hence, TME promotes Th1 cells reprogramming to Th17 cells, then to T_regs_ under intratumoral high lactate level that also suppresses IL-2 signaling (342). The increase in the glutaminolysis in tumor cells also deprives infiltrated T cells of glutamine, further compromising their growth and proliferation (**Figure 8**) (344). The glutamine-deficient TME reduces cytosolic α-KG in Th1 cells supporting their differentiation to T_regs_ (345). The glutaminase (a key enzyme involved in glutaminolysis) genetic deletion or glutamine uptake blockade in tumor cells increases TME glutamine and upregulates T cell infiltration (128, 346). The glutaminolysis is linked to polyamine biosynthesis via a Myc-dependent metabolic pathway in T cells (347). Hence, immunometabolic reprogramming among tumor-infiltrated T cells is governed by TME, including the hypoxia and lactate level.

HIF-1α during hypoxia induces the PDL-1 (CD274) expression in tumor cells, DCs, TAMs, and MDSCs to support immunosuppressive TIME (188). For example, PD-1^+^ CD8^+^T cells in TIME are most immunodysfunctional due to the mitochondria loss (348). The mitochondria loss affects their oxidative (TCA cycle, FAO, and OXPHOS) and membrane potential (ROS and ATP production) due to PPAR-γ coactivator 1α (PGC1α) loss, which programs mitochondrial biosynthesis by Akt signaling (348). B-lymphocyte-induced maturation protein 1 (BLIMP1) activation causes PGC1α loss. The PGC1α loss increases ROS production that, through phosphatase inhibition and the consequent activity of NFAT, promotes T cell exhaustion through mitochondrial dysfunction and loss (349–351). The mitochondrial mass loss in CD8^+^T cells of TIME correlates well with PD-1 expression. Thus, the PD-1/PDL-1 interaction in TIME suppresses T cell immune response governed by their metabolic stage or alters T cell immunometabolism responsible for immunosuppression (142). The increased lipolysis of endogenous lipids and FAO among PD-1^+^CD8^+^T cells continuously exposed to PDL-1-expressing cells survive longer to support the immunosuppressive TIME. These immunosuppressive CD8^+^T cells highly express CPT1A and the adipose triglyceride lipase (ATGL), the lipolysis marker glycerol, and the release of FAs (142). On the other hand, the T_regs_ PD-1 engagement with PDL-1 promotes FAO and mitochondrial OXPHOS to fuel their energy requirement in the presence of TGF-β (142, 352).

TGF-β suppresses PI3K-mediated mTOR signaling and inhibits glucose transporter and *hexokinase 2* (*Hk2*) expression that favors OXPHOS in induced-T_regs_ (iT_regs_). PD-1 reduces the TGF-β threshold for its immunosuppressive action, including the T_reg_ development and function in the TIME (353). Reduced TGF-β signaling via TGF-β type 1 receptor (TβR1) is crucial for T cell activation and associated immune response (354). Hence, the increased TGF-β in TME and TIME suppresses antitumor T cell immune response through metabolic reprogramming. The details of PD-1 signaling mediated T cell immunometabolic reprogramming responsible for T cell immunosuppression are discussed elsewhere (141). Blocking PD-1/PDL-1 signaling restores glucose in TME, which permits T cell glycolysis and IFN-γ production as an antitumor immune response (78). However, blocking PD-L1 directly in tumors inhibits their glycolysis via suppressing mTOR signaling and glycolysis enzymes (78). Tumor and immune cell-secreted and expressed molecules create a T cell-mediated immunosuppressive TIME in the TME to support tumor growth, proliferation, and metastasis *via* immunometabolic reprogramming.

### 4.6 B cells in TIME and their immunometabolic reprogramming

**Figure 9:**
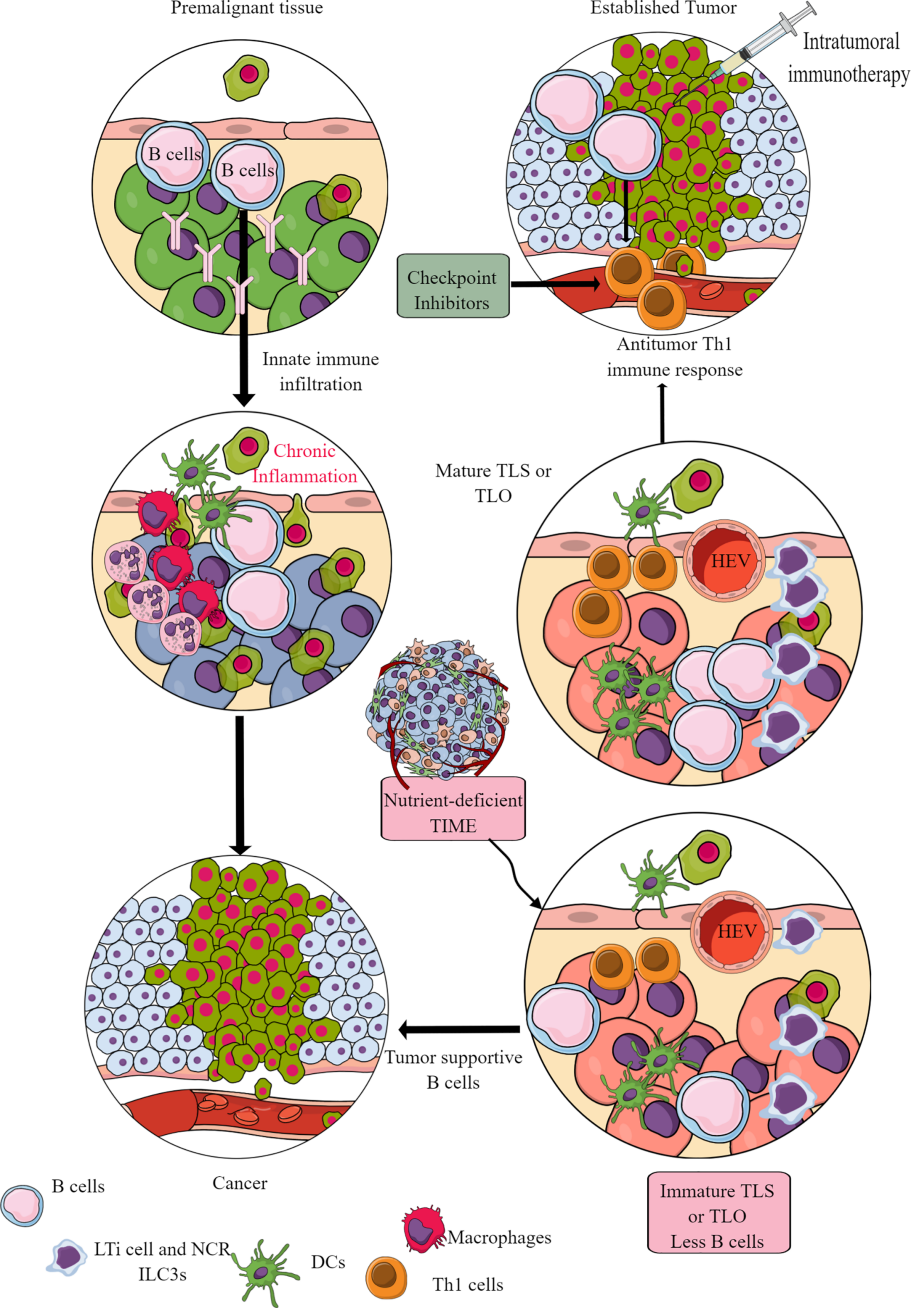
B cells in TIME. B cells in the premalignant tissue are the first immune cells to send signals to innate immune cells. This causes chronic inflammation. Unresolved chronic inflammation is linked to several cancers, including lung, breast, and colorectal cancers. However, in established tumors, B cells serve as antitumor immune cells and support intratumoral immunotherapies and Th1 immune response. However, only mature B cells perform antitumor functions, and their maturation occurs in the TLS or TLOs. The nutrient-deprived TME or TIME does not support TLS maturation, and immature B cells and B_regs_ increase immunosuppressive TIME. See text for details.

Murine cancer models have indicated the role of B cells in tumor pathogenesis and immunity, including their regulatory role in innate immune cell infiltration in the premalignant tissue to promote chronic inflammation, which promotes epithelial carcinogenesis (**Figure 9**) (355, 356). For example, antibodies released from activated B cells in premalignant tissues fuel chronic inflammation through Fcγ receptor (FcγR)-dependent innate immune cell infiltration into the preneoplastic and neoplastic TME (**Figure 9**). Hence, B cells have been shown to promote cancer through promoting early malignancy via supporting chronic inflammation. However, in established tumors, B cells act as antitumor immune cells by promoting IFN-γ secreting Th1 immune cells, which are crucial for generating an adequate antitumor immunity in response to checkpoint inhibitors (357, 358). Even, intratumoral immunotherapy success depends on B and T cell collaboration (359, 360). In humans, intratumoral B cells are good prognosis markers for different cancers (361). However, the clonal diversity among infiltrated B cells affects survival of patients with cancer depending on type (362–364). For example, in TME or TIME, intratumoral B cell number highly depends on tertiary lymphoid structures (TLSs), as tumors without TLS have low B cell numbers (365, 366). Furthermore, B cell maturation, selection, and expansion occur in the mature TLS of tumor tissue that determines their antitumor (367, 368).

The mature TLS B cells increase antitumor T-cell activity in TIME and the responsiveness of tumors to immunotherapies (**Figure 9**) (368). On the other hand, B cells in immature TLSs do not have potent antitumor action. Instead, they become tumor-supportive (**Figure 9**). The B cell numbers, including the presence of switched memory B cells in tumor TLSs, guide the success of potential tumor immunotherapy and the associated patient survival (365). The details of B cells in TME and TIME are discussed elsewhere (361, 369–371). However, TLS maturation depends on the availability of extracellular ATP molecules (including the microbe-derived ones), which use ILC3-driven (IL-22, TNF-α, IL-8 and IL-2) and colony-stimulating factor 2 (CSF2)-dependent axis to induce the monocyte to macrophage transition in TIME (372, 373). These NCR^+^ILC3s are in higher numbers in the early stages (stage 1 or 2) than in later tumor stages, and their presence directly correlates with the density of TLSs in TIME (373). For example, gut microbiota may influence the efficacy of tumor immunotherapy via many immunomodulatory mechanisms, including the secretion of metabolites supporting the development and maturation of TLSs in TIME (374). Hence, a nutrient-competitive TME does not support the maturation of TLSs in TIME to escape from B cells and other immune cell-based antitumor immunity (**Figure 9**).

B cells are divided into B1 B cells, conventional B2 cells, and marginal zone B (MZB) cells. MZB cells have an innate-like function and are present mainly in the spleen along with LNs and blood to take care of blood-borne pathogens and circulating antigens or foreign particles (375, 376). Out of B1 (form in fetal life and then depend on self-renewal in adult life) and B2 B cells (constantly keep developing in BM), B1 B cells are more dependent on OXPHOS and glycolysis and are more active at the resting stage than B2 B cells (377–379). In addition, B1 B cells acquire external lipids as lipid droplets. Furthermore, B1 B cells have a unique immunometabolic programming that depends on their location and specific functional properties as autophagy-deficient B1-a B cells down-regulate critical metabolic genes and accumulate dysfunctional mitochondria (379). Hence, the autophagy gene Atg7 is crucial to maintain their immunometabolic status to support their high proliferative and secretary functions.

Non-proliferative naïve B cells depend on OXPHOS due to the glycogen synthase kinase 3 (GSK3) activity required to maintain their metabolic quiescence and prevent proliferation (380). However, tumors have insufficient naive non-proliferating B cells (371). The germinal center (GC) B cells have different immunometabolic requirements depending on their location in the light zone (LZ) and dark zone (DZ). For example, in mature GCs (the microanatomical sites of antibody diversification (B cell clonal expansion) and affinity maturation), the DZ has large and mitotically active proliferating B cells (centroblasts) undergoing somatic hypermutation (SHM). These DZ B cells depend on glycolysis for the energy demand and differentiate into LZ B cells (381). Whereas the LZ of the GC contains a large portion of infiltrating and non-proliferative quiescent naïve B cells, which are also called centrocytes and compete for antigen presentation to follicular helper T (T_fh_) cells mainly depend on OXPHOS (382, 383). However, LZ B cells expressing BCR and CD40 are rewired to highly express c-Myc, stimulating mitochondrial biosynthesis and genes required for glycolysis, promoting their re-entry to the DZ of the GC (384–386). These c-Myc-expressing centrocytes also express HIF-1α to support anaerobic glycolysis (387).

Memory B cells depend on OXPHOS for their metabolic demand. In contrast, plasma cells (PCs) or antibody-secreting B cells depend on OXPHOS and other carbon-utilizing metabolic pathways, including the TCA cycle, and nucleotide biosynthesis (PPS or PPP) that supports ribosome synthesis or ribogenesis, but not glycolysis (388–390). This glucose deprivation of PCs does not affect their humoral functions, but OXPHOS and glutaminolysis inhibition impairs their growth and differentiation. Hence, B cell activation requires considerable mitochondrial remodeling due to extensive OXPHOS. In addition, long-lived plasma cells (LLPCs) also depend on amino acid metabolism (glutaminolysis) and autophagosome formation (391, 392). Notably, PC metabolic reprogramming may also be affected by other factors, including the type of antibody production, location, and other metabolites (vitamins). For example, vitamin B1 supports the TCA cycle in Peyer’s patches in IgM-producing PCs without affecting IgA production (393). The tumor-infiltrating IgM memory B cells and switched memory B cells (IgG- and IgA-producing PCs) are present in different cancers, including breast cancer (BC), renal cell carcinoma (RCC), and head and neck squamous cell carcinoma (HNSCC) (394–396). The GC B cells, plasmablasts, and plasma cells are present in non-small cell lung cancers (NSCLCs), RCCs, HNSCC, and ovarian and prostate cancers (367, 395–397).

In TME or TIME infiltrated B cells under the influence of IL-6, IL-1β, IL-12p35, and low oxygen (tumor-promoting molecules), which polarize to regulatory B cells (B_regs_), producing TGF-β, granzyme B (GZMB), IL-10, and IL-35, which promote tumor growth and metastasis (398–402). The metabolic reprogramming among B_regs_ in TIME is unclear, but IL-10 secretion depends on glucose influx-dependent OXPHOS, PPP, amino acid metabolism, and oxygen level in the TME. Also, a balance between B_regs_ and PCs derives potential antitumor immunity during pancreatic cancer (403). However, IL-35 in TME breaks this balance and stimulates the STAT3-paried box 5 protein (PAX5, a transcription factor crucial for B cell differentiation) complex, upregulating B cell lymphoma 6 (BCL6, a transcriptional regulator) in naive B cells. BCL6 inhibition in tumor-educated B cells reverses dysregulated B cell differentiation and stimulates the intra-tumoral accumulation of PCs and T_effs_. This renders pancreatic tumors sensitive to anti-PD-1 blockade (403). Hence, B cell metabolic reprogramming in the TME or TIME alters their antitumor action and promotes their polarization to tumor-supportive B_regs_.

## 5 Targeting immunometabolic reprogramming in cancer

The dendrimer-mediated nanomedicine-based therapeutic targeting of TAM-specific mitochondria in glioblastoma has stimulated their anticancer function (404, 405). Also, targeting TAMs of pancreatic ductal adenocarcinoma (PDA) to block the pyrimidine metabolites’ release, including deoxycytidine, sensitizes tumors to the anticancer drug gemcitabine (a pyrimidine anti-nucleoside) (406). The pyrimidine synthesis in M2 macrophages occurs in response to the increased FAO and TCA cycle (406). Also, serine metabolism is crucial for M1 to M2 macrophage polarization to support immunosuppressive TIME. Serine depletion, either by inhibiting phosphoglycerate dehydrogenase (PHGDH, crucial in the serine biosynthesis pathway) or by exogenous serine and glycine restriction, robustly enhances the polarization of M1 macrophages with antitumor potential along with suppressing M2 macrophages (407). Serine metabolism inhibition in macrophages increases the insulin-like growth factor-1 (IGF1) expression via decreasing the S-adenosyl methionine (SAM)-dependent histone H3 lysine 27 trimethylation. IGF1 then stimulates p38-dependent Janus kinase or JAK–STAT1 axis, promoting M(IFN-γ) or M1 polarization and suppressing M(IL-4)) or M2 macrophages (407). Hence, targeting macrophage metabolism in different cancers can increase the efficacy of available chemotherapies.

Also, targeting glutamine metabolism in TME blocks the immunosuppressive effects of MDSCs, induces their activation-induced cell death (AICD), and the MDSC transition to antitumor M1 macrophages (138). Glutamine metabolism inhibition, specifically to tumor and myeloid cells with a prodrug called 6-diazo-5-oxo-L-norleucine (DON), decreases CSF3 level in TME that blocks MDSCs recruitment and induces immunogenic cell death, promoting the recruitment of M1 macrophages. Targeting glutamine metabolism also inhibits the tryptophan metabolism generating immunosuppressive kynurenine metabolites (138). However, glutamine deprivation in CD8^+^T cells of hepatocellular carcinoma (HCC) induces their apoptosis due to mitochondrial dysfunction (408). Hence, cell-specific glutamine metabolism targeting specifically in the TME/TIME may serve as a potential immunometabolism regulatory approach. However, lactate treatment increases the stemness of CD8^+^T cells to augment their antitumor action by inhibiting histone deacetylase (HDAC) activity that acetylates H3K27 of the transcription factor 7 (Tcf7) super-enhancer locus causing its increased gene expression (409). Furthermore, the adoptive transfer of CD8^+^T cells treated *in vitro* with lactate show an increased antitumor action. Hence, adoptive transfer of oncometabolites’ treated T cells may serve as immune cell-based therapeutics for cancer due to their epigenetic modification and resistance development to harsh TME. However, further studies are needed in this direction.

The CD28-mediated co-stimulation among tumor (ccRCC) infiltrated CD8^+^T cells has restored their defective glycolysis and mitochondrial oxidative metabolism by upregulating Glut3 (410, 411). However, an early study indicated that glycolysis does not support long-term memory CD8^+^T cell formation and their antitumor action (412). Hence, it becomes crucial to explore these effects related to glycolysis in naïve CD8^+^T cells or tumor-infiltrated CD8^+^T cells to better design immunometabolic reprogramming approaches specific to different cancers. CD47 regulates CD8^+^ T cell activation, proliferation, and fitness in a context-dependent manner, including cancer (413). So, it will be novel to understand the impact of CD47 engagement on glycolysis in CD8^+^T cells in TIME or homeostasis. For example, CD47 blockage on CD8^+^T cells mediates immunogenic tumor destruction (414, 415). Furthermore, the decreased CD47 expression on cancer cells increases macrophage infiltration in tumors with an enhanced potential to phagocytose cancer cells (416). CD47 expression increases in TME in response to IL-18 released from macrophages during chemotherapy (doxorubicin). IL-18 upregulates L-amino acid transporter 2 (LAT2) expression in tumor cells, enhancing leucine and glutamine uptake. Glutamine and leucine are two potent mTORC1 signaling stimulators. Thus, increased cellular leucine levels and glutaminolysis activate mTORC1 signaling, which by c-Myc activation, induces CD47 transcription and expression (416). Hence, CD47 blocking in CD8^+^T cells and tumor cells may increase tumor clearance and patient survival through metabolic alteration of tumor and immune cells.

Additionally, glutarate administration reduces the tumor burden by increasing CD8^+^T cells in the TME and systemic circulation and their antitumor function by immunometabolic reprogramming (417). The glutarate reprograms CD8^+^T cell immunometabolism responsible for their cytotoxic function, involving a post-translational modification of the pyruvate dehydrogenase E2 (PDHE2) subunit of the PDH complex (PDHc). The PDHc glutarylation induces a rapid pyruvate conversion to lactate and increased glycolysis in CD8^+^T cells to reprogram their antitumor function (417). Furthermore, the magnesium (Mg^2+^) treatment increases the co-stimulatory function of leukocyte function-associated antigen-1 (LFA-1) on CD8^+^T cells to exert their cytotoxic action against tumor cells via different mechanisms, including immunometabolic reprogramming (418, 419). CAR-T cells also exert an improved and more extended antitumor function upon Mg^2+^ supplementation. Notably, TME has less available Mg^2+^ for immune cells, including CD8^+^T cells, due to its high usage by tumor cells. Hence, intratumoral Mg^2+^ supplementation improves antitumor TIME to fight against tumors and improves CAR-T cell-based immunotherapy.

L-arginine availability to T cells increases their survival by immunometabolic reprogramming (transition of glycolysis to OXPHOS). It promotes their differentiation to central memory-like T cells with anti-tumor activity without inducing mTOR signaling (420). L-arginine increases T cell survival in TME through targeting transcriptional regulators bromodomain adjacent to the zinc finger domain 1B (BAZ1B) or Williams syndrome transcription factor (WSTF), PC4 and SFRS1 interacting protein 1 (PSIP1), and translin (TSN) (420). The mitochondrial arginase 2 (Arg2) depletion in CD8^+^T cells increases their survival and antitumor action (421). The CD8^+^T cell-specific Arg2 inhibition synergizes the antitumor action of PD-1 blocking checkpoint inhibitors. Thus, l-arginine depletion in the TME or TIME by tumor cells and myeloid suppressor cells due to the activation enzymes (Arg1 and iNOS2) compromises an efficient antitumor action of T cells, including CD8^+^T cells to clear tumor cells (422). The use of genetically modified bacteria (*Escherichia coli* Nissle 1917 strain) or ECN that utilizes ammonia to synthesize L-arginine in tumors has increased antitumor T cell infiltration in TME or TIME to clear the tumor (423, 424). This genetically modified bacteria used as bacterial anticancer therapy (BAT) works synergistically with PD-1 blockers to clear tumors. Hence, emerging immunometabolic reprogramming targeting different cancers has a better future as a specific-immune cell-based tumor targeting and synergizing the available checkpoint inhibitors.

## 6 Future perspective and conclusion

The immune system is key to checking the induction, development, growth, and metastasis of cancer. Immunometabolic reprogramming among immune cells governs their stimulatory and inhibitory immune functions depending on the stimuli and tissue environment. Thus, it has become crucial to understanding immunometabolic reprogramming and its governing factors in TIME. The development of a robust immunosuppressive TIME has become a landscape for tumor growth, proliferation, and metastasis. For example, increased lactate levels in TME or TIME induce immunosuppressive immunometabolic reprogramming and block the antitumor function of immune cell-based immunotherapies (adoptive T cell therapies) and checkpoint inhibitors (425, 426). LDHA inhibitor (GSK2837808A) has improved the antitumor activity of CD8^+^T cells via altering their immunometabolic reprogramming responsible for their exhaustion and apoptosis (426). Furthermore, TME or TIME lactate levels can be lowered using MCT1 and MCT4 lactate transporter inhibitors (AZD3965) to improve the existing immune cell-based therapies (427–429).

The increased lactate accumulation in TME or TIME occurs due to overwhelming glycolysis in tumor cells (426). Thus, tumor cell-specific glycolysis can also be a therapeutic approach that directly targets tumor cells, and will also increase the efficacy of immune cell-based immunotherapies and checkpoint inhibitors via decreasing the TME lactate levels (430–433). Fumarate accumulation in TME also inhibits B cell function via covalent inhibition of a tyrosine kinase LYNN of the B cell receptor (BCR) signaling pathway (434). The fumarate deposition blocks BCR signaling-mediated antitumor action, including antibody production and cytokine release. Additionally, fumarate has other tumor-supportive effects by altering different immune cells, but its impact on their immunometabolic reprogramming remains to study (435). Hence, targeting tumor cell-specific glycolysis and lactate and fumarate accumulation in TME indirectly enhances the antitumor action of immune cells by immunometabolic reprogramming. Many metabolic inhibitors with potential to clinical translation are at different clinical trial stages (II and III), which can be used to reprogram TME immunometabolism (23, 25).

Calcium carbonate (CaCO_3_) nanoparticles coated with 4-phenylimidazole (4PI) inhibit IDO1 to increase the radiotherapy efficacy (436). These nanoparticles are called acidity-IDO1-modulation nanoparticles (AIM NPs), which instantly neutralize protons (H^+^) and release 4PI to inhibit the immunosuppressive IDO1 activity in the TME. Thus, AIM NPs reinforce the radiotherapy via modulating the immunosuppressive metabolic reprograming in the TME. Another nanoparticle-based approach during low dose radiotherapy has increased ICIs (PD-1/PD-L1 blockers) efficacy via reprograming immunosuppressive TME immunometabolism in triple negative breast cancer (TNBC) patients (437). This approach involves scavenging the reduced nicotinamide adenine dinucleotide phosphate (NADPH) inside tumor cells by developing the nanomolecule (BMS202@HZP) targeting hypoxia and PD-1/PD-L1 interaction during low dose radiotherapy against TNBC (437). Along with conventional nanomedicine, thermal-immuno nanomedicine is emerging as potential antitumor therapy (438–440). Hence understanding and developing nanomedicine-based approaches specifically targeting TME immunometabolism have a bright future for tumor immunotherapy. For instance, understanding metabolic reprograming, including immunometabolism can rewire radio- oncology for better therapeutic ratio or outcome (441). We need further studies in this direction.

Aging is one of several predisposing factors for cancer as it alters immune cell functions via inducing altered immunometabolism (442, 443). For example, the B cells of older people show a significant reduction in their OXPHOS compared to glycolysis (444). Also, T cells isolated from older adults exhibit decreased glycolysis and OXPHOS but increased mitochondrial ROS generation, indicating an impaired mitochondrial function (442). Aging-associated immunometabolic reprogramming among older adults induces a stage of chronic inflammation that may serve as cancer predisposing factor. Thus, the immunometabolic profile of aged people may indicate their future risk for cancer. Spermidine, a polyamine considered an antiaging molecule enhances the antitumor action of CD8^+^T or nanobody-based CAR-T cells (Nb CAR-T) cells via immunometabolic reprogramming that increases IFN-γ and IL-2 production (445).

OVT is an emerging area to convert cold tumors to hot tumors or TME through reprogramming immunosuppressive TIME to pro-inflammatory antitumor immunity when used alone or with available checkpoint inhibitors (446–449). However, how OVT modulates the immunometabolic reprogramming among specific immune cells of TIME is an exciting research area to delineate. Also, immunometabolism has emerged as a novel way to target specific immune cell populations in diverse diseases, including sepsis, autoimmunity, and other infectious diseases. The information discussed in the present article specifies that the immunometabolic reprogramming among infiltrated immune cells alters in TME or TIME and needs great attention as it diverts immune cells’ normal antitumor function to support tumor growth and metastasis. Hence, immunometabolic reprogramming is another cancer hallmark with significant therapeutic potential based on cancer stages and immune cell population. Thus, studying cancer-associated immunometabolic reprogramming will help to design better immune cell-based therapies, BATs, and OVTs in the future.

## Author contributions

VK has developed the idea, wrote the article and conceptualized and developed the figures. JS has done the proofreading and final edits.
